# Hepcidin Gene Co-Option Balancing Paternal Immune Protection and Male Pregnancy

**DOI:** 10.3389/fimmu.2022.884417

**Published:** 2022-04-22

**Authors:** Wanghong Xiao, Zelin Chen, Yanhong Zhang, Yongli Wu, Han Jiang, Huixian Zhang, Meng Qu, Qiang Lin, Geng Qin

**Affiliations:** ^1^ Southern Marine Science and Engineering Guangdong Laboratory (Guangzhou), Guangzhou, China; ^2^ CAS Key Laboratory of Tropical Marine Bio-resources and Ecology, South China Sea Institute of Oceanology, Chinese Academy of Sciences, Guangzhou, China; ^3^ University of the Chinese Academy of Sciences, Beijing, China

**Keywords:** seahorse, paternal immune care, male pregnancy, gene co-option, hepcidin

## Abstract

Viviparity has originated independently more than 150 times in vertebrates, while the male pregnancy only emerged in Syngnathidae fishes, such as seahorses. The typical male pregnancy seahorses have closed sophisticated brood pouch that act as both uterus and placenta, representing an excellent model system for studying the evolutionary process of paternal immune protection. Phylogenetic analysis indicated that the hampII gene family has multiple tandem duplicated genes and shows independent lineage-specific expansion in seahorses, and they had the highest ratio of nonsynonymous substitutions to synonymous substitutions (dN/dS) in the seahorse phylogenetic branch. The expression levels of hampIIs in the brood pouch placenta were significantly higher during pregnancy than non-pregnancy. Both LPS stimulation test *in vivo* and cytotoxicity test *in vitro* proved the immunological protection function of hampIIs against pathogen infection in seahorse. Besides, seahorse hampII peptides exhibit weaker antibacterial function, but stronger agglutination and free endotoxin inhibition. We assumed that the modified immunological function seemed to be a trade-off between the resistance to microbial attack and offspring protection. In brief, this study suggests that the rapid co-option of hampIIs contributes to the evolutionary adaption to paternal immune care during male pregnancy.

## Introduction

In viviparous animals, maternal immune protection of offspring is common during pregnancy, while the paternal immune protection is negligible in most vertebrates (1–3). Viviparity has an independent origin and appears more than 150 times in vertebrates, while male pregnancy only emerges once in Syngnathidae fishes (4). Seahorses, the iconic species in the Syngnathid family with typical male pregnancy, have males with closed sophisticated brood pouch that act as the uterus during pregnancy, which provide protection, osmoregulation, gas exchange, and nutrition to the embryos (5, 6). The brood pouch of male seahorse undergoes remodeling and even develop placenta during pregnancy (5, 7). Previous studies had figured the trade-off between immunological tolerance and embryo rejection along with the evolution of unique male pregnancy in the Syngnathid family (8, 9). Therefore, we assumed that the seahorses may serve as an excellent model system for studying the evolutionary process of paternal immune protection.

**Figure 1:**
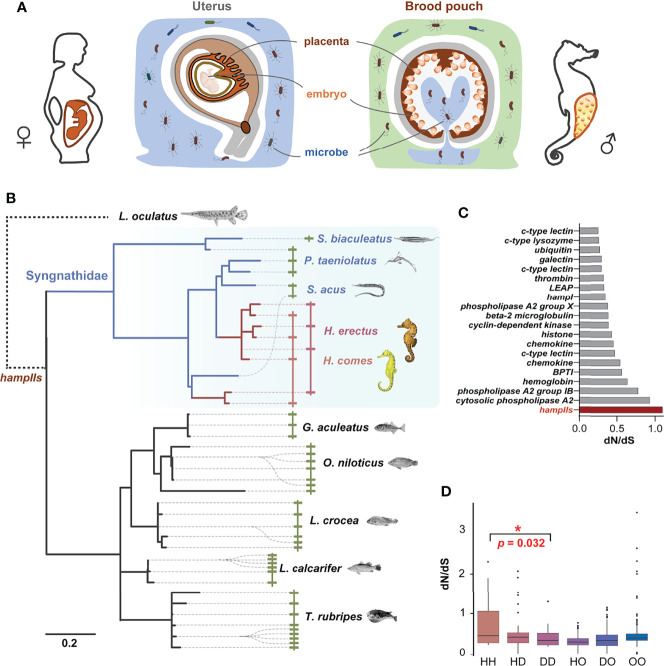
HampII gene co-option in male pregnancy in seahorse. **(A)** Seahorses have a sophisticated brood pouch with a placenta structure inside, which is functionally similar to the uterus and placenta in mammals, and plays an important role in preventing uncontrolled bacterial growth in the closed nutrient-rich environment, without damaging the developing embryos during pregnancy. **(B)** Phylogenetic analysis (Bayesian tree) showed taxonomic- or lineage- specific expansions in hampII genes (left), and synteny analysis demonstrated that hampII genes contained tandem duplicated sequences in the same scaffold (right). HampIIs in Syngnathidae were highlighted in blue background, seahorse hampIIs in red line, and other Syngnathidae hampIIs in blue line. **(C)** The dN/dS value of the seahorse branch of the hampII genes was the highest amongst all the antimicrobial peptides. Seahorse hampIIs were denoted in red. **(D)** The pairwise dN/dS values between hampII gene pairs was significantly higher in seahorses than other Syngnathidae (H, seahorses; D, Syngnathidae but not seahorse; O, other teleost species) (*, *p <*0.05).

It is especially critical to maintain the parent’s immunological vigilance towards invading pathogens to protect both parents and infirm embryos of seahorse during pregnancy. The non-sterile sea water always filled into the brood pouch along with female’s eggs during mating, and male seahorses have to prevent bacteria burst in the nutrient-rich environment in the closed brood pouch during pregnancy (5, 10). A pathogen-limited environment in the closed seahorse brood pouches was speculated to be maintained by a balance between the paternal immune molecules and commensal microbiota (**Figure 1A**) (10, 11). Compared to mammal’s placenta, the thin tissue of placenta in seahorse’s brood pouch seems be weak in functioning as an immunological barrier between the father and offspring (5, 11), so, the excessive immune response might recognize and attack the embryos as foreign, leading to abortion or death (12). Therefore, we speculated that a trade-off between microbial attack and offspring protection in the brood pouch should be critical for male seahorse’s successful pregnancy.

Previous studies have figured that prominent antimicrobial peptides (AMPs) in seahorse brood pouches have positive immunoprotective effects and might contribute to successful embryo incubation during pregnancy, similar to those in mammalian mothers (13–15). Hepcidin is a small cysteine-rich antimicrobial peptide, including two types: hepcidin I and II. The hepcidin I (HampI) is a key regulator of iron metabolism for vertebrates, while hepcidin II (HampII) is considered to mainly function as immune defence in fish (16, 17). HampII only present in teleost fishes with multiple copies, which is a result of genome duplications and tandem duplications (18, 19). HampIIs are thought to play essential roles in evolutionary response to extreme and diverse water environments through gene co-option (19). Gene co-option, mostly *via* gene duplication and functional divergence of paralogs, changes the expression patterns and functions of the encoding proteins, contributing to developmental and physiological novelties (20–22). For instance, duplication and diversification of hampII genes play important roles in evolutionary response to the cold and oxygen-rich water in Antarctic notothenioid fishes (23). And immunological function diversity among hampII gene copies, have allowed large yellow croaker to adapt to diverse environmental conditions (19). We assume that gene co-option of hampIIs might occur in seahorse so as to play unique immune roles in the brood pouch during male pregnancy.

In the present study, we found that the recent lineage-specific gene expansion and rapid molecular diversification of newly acquired hampII copies, led to novel spatiotemporal expression profiles and modified antibacterial functions, thereby contributing to the evolutionary adaptation to specific paternal immune care in seahorses during male pregnancy.

## Results and Discussion

### Genomic Evolution of HampII Gene Families in Seahorses

Four *hampII* genes (*HehampII-1,2,3,4*) were identified from the lined seahorse genome and cloned from the cDNA. We also searched for homologous *hampII*s from the genome of five other Syngnathidae and five other teleost species (**Figure 1B** and **Table S1**). Although the largest *hampII* families (11 genes) were found in fugu, and the *hampII* families in seahorses (*Hippocampus* genus) are more than those in other Syngnathidae fishes (**Figure S1**). All the structures of hampII precursor proteins are conserved, including the signal peptide, prodomain, and mature peptide, while the sequences of seahorse hampIIs mature peptides varied remarkably from other teleost fishes including their Syngnathidae relatives (**Figure S2**). What’s more, the molecular evolution analysis based on a total of 246 antimicrobial peptide genes among two seahorse species and other eight teleost fishes figured that the *hampII* gene family had the highest ratio of nonsynonymous substitutions to synonymous substitutions (dN/dS) in the seahorses’ most recent common ancestor (MRCA), indicating rapid sequence evolution rates in seahorses’ *hampII* genes compared to other antimicrobial peptides (**Figure 1C** and **Table S2**).

Interestingly, phylogenetic analysis indicated that the *hampII* family has multiple tandem duplicated genes and shows independent lineage-specific expansion in most teleost species, as well as during the evolution of Syngnathidae (**Figure 1B**, **Figure S1** and **Figure S3**). Synteny analysis demonstrated that all *hampII* genes were tandem duplicated at the same genomic locus in all species. The duplicated copies, freed from purifying selection, are expected to evolve rapidly. It’s worth noting that evolutionary rates of Syngnathidae *hampII*s were faster than that of other teleosts (**Figure 1B** and **Figure S3**), a recent expansion occurred in the ancestor of the seahorse but not in those of the other Syngnathidae (**Figure 1B** and **Figure S3**), and the dN/dS values between the seahorse *hampII* genes were significantly higher than those between other Syngnathidae (**Figure 1D**). These high variations in seahorse *HehampII* genes, especially located in the mature peptide region (**Figure S2**), might be conducive to new functions (24). Previous studies had figured that the frequent species or lineage-specific expansion of *hampII* genes in marine fishes were in line with evolutionary adaptation to the complexity and challenges of environmental conditions the fish encounters and copes with during its life history (19, 24). For example, the reduction to four cysteines from commonly eight cysteines in the *Antarctic notothenioid hamp* genes were supposed to increase the flexibility of the hairpin structure so as to adapt to the cold temperature (23). The *hampII* genes of large yellow croaker (*Larimichthys crocea*) were functionally diverse, involving in antibacterial or antiviral activity, so as to adapt to diverse environmental conditions (19). Therefore, the lineage-specific expansion and rapid evolution of the *hampII* genes in the seahorses might have promoted gene co-option and associated trait novelty, namely the immunological function.

### Gene Expression Patterns of HehampII Family During Seahorse’s Breeding Cycle

**Figure 2:**
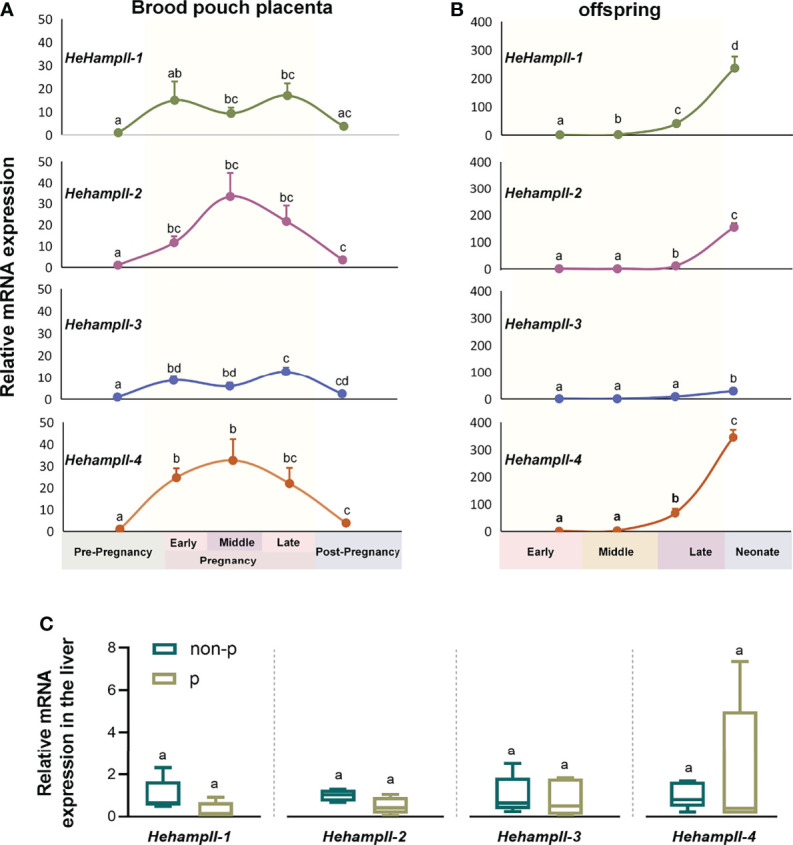
The expression of HehampII genes in the brood pouch placenta **(A)** of lined seahorse (*Hippocampus erectus*) was higher during pregnancy, whereas the highest expression in the offspring **(B)** was observed at the post pregnancy period when the new-borns were just released from the closed brood pouch to the water environment (n ≥ 5, mean ± SEM). **(C)** No significant differences were detected in the expression of any seahorse HehampIIs in the liver between pregnant (p) and non-pregnant seahorses (non-p). Different lowercase letters indicate significant differences between them (*p* < 0.05).

In order to verify whether *HehampII* genes play a role during male seahorse pregnancy, we detected the gene expression patterns during seahorse’s breeding cycle. Firstly, the qPCR results showed that all *HehampII* genes are widely expressed in most organs of lined sehaorse, although the highest expression were detected in the liver (**Figure S4**). It is inconsistent with previous report in large yellow croaker, in which five hampII genes had dramatically different expression patterns (19). Interestingly, we found that the expressions of *HehampII* genes were dynamic in both the father’s brood pouch placenta and offspring during pregnancy. During pregnancy, the expression levels of all *HehampII* genes in the brood pouch placenta significantly increased, peaked at about 20-30 times than pre-pregnancy period, and then decreased sharply after parturition (**Figure 2A**). Meanwhile, the expression levels of all *HehampII* genes in the offspring began to rise until the late pregnancy period, and then increased quickly in the newborn neonate when they were just released from the closed brood pouch to the water environment (**Figure 2B**). In contrast, no significant changes were observed in seahorse liver during seahorse’s breeding cycle (**Figure 2C**). These specific expression profiles of *HehampII* genes during male pregnancy, which are similar to some other immune genes reported before, such as TLR2 (25), α2M (26), and C-type lectin (13), strongly suggest that *HehampII* genes play some roles in the brood pouch during male pregnancy.

### Immunoprotective Effects of Seahorse Hehamp *In Vitro* and *In Vivo*

**Figure 3:**
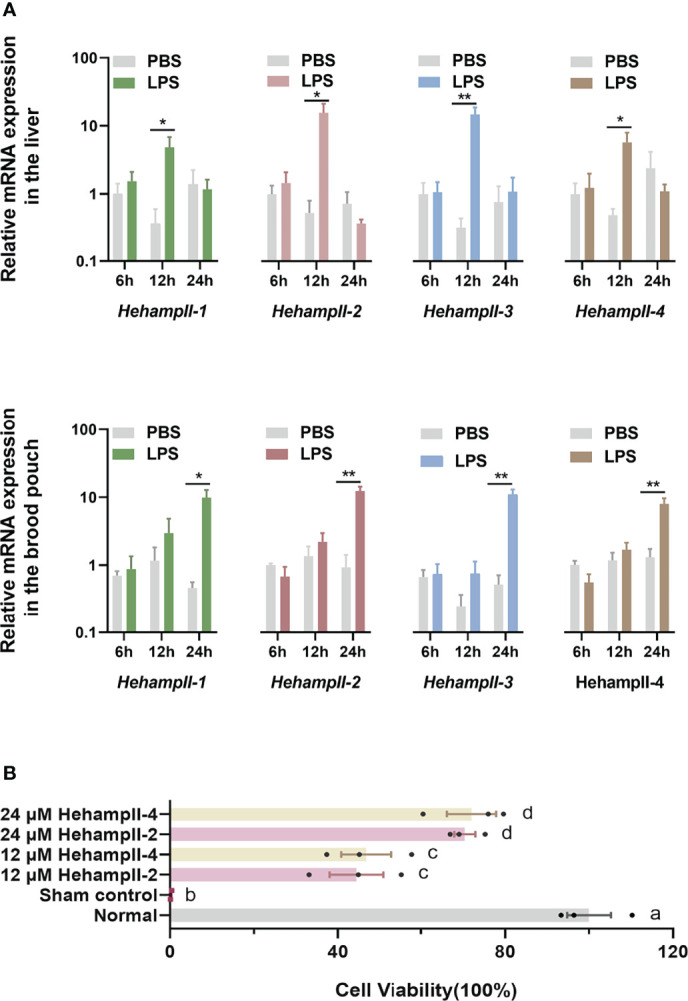
**(A)** *In vivo*, relative expression of seahorse HehampIIs in the placenta of the brood pouch and liver were upregulated in response to the lipopolysaccharides (LPS) challenge, PBS: phosphate-buffered saline. (mean ± SEM, n=5; *, *p <*0.05; **, *p <*0.01) **(B)** *In vitro*, the two seahorse HehampII mature peptides could rescue the death of seahorse embryonic cell caused by supernatant of *Vibrio.* Different lowercase letters indicate significant differences between them (mean ± SEM, n=3; *p* < 0.05).

Hepcidins including both HampI and HampIIs were supposed to mainly perform an antimicrobial role in fish (27). LPS is a component of the outer wall of gram-negative bacteria and can activate a number of immune-related genes and signaling pathways (28). Hepcidins from the redbanded seabream (*Pagrus auriga*) (29) and mudskipper (*Boleophthalmus pectinirostris*) (30) were increased post LPS challenge, suggested their immune protective role in fish. Hepcidin from caspian trout (*Salmo caspius*) upragulated after LPS stimulation, and it’s synthetic mature peptide exhibited antibacterial activity to several strains (31). To verify the function of HehampIIs in the brood pouch of seahorse, Lipopolysaccharide (LPS), a common pathogen-associated molecular pattern (PAMP) used for mimicking pathogenic infection, were injected into the brood pouch. The *in vivo* test showed significantly upregulated expression of all the *HehampII* genes in response to LPS challenge at 24 hours post injection (dpi) in the seahorse brood pouch, meanwhile, similar gene expression responses were also observed in the liver at earlier time point (12 h) (**Figure 3A**). These results proved that *HehampII* genes are indeed involved with the immune response in the brood pouch of seahorses.

Considering the close contact between the simple-structured placenta and thin-chorion embryos (11), these almost perfect transform of *HehampII* expression levels between the brood pouch and the incubated embryos implied that seahorse father might produce HehampII peptides as immune substances to protect the early embryos lack of complete immunity (**Figures 2A, B**). In order to test this assumption, cytotoxicity test *in vitro* were finished using the synthesised mature polypeptides of HehampII-2 and HehampII-4, respectively, encoding by the two seahorse lineage-specific hampII genes (*HehampII-2*, *HehampII-4*), which were also the most highly expressed hampII genes in the brood pouch during pregnancy (**Table S4** and **Figure 2**). During the cytotoxicity test *in vitro*, the HehampII peptides at different concentration (12 μM and 24 μM) were added into the seahorse embryonic cells along with supernate of pathogenic *Vibrio parahaemolyticus*, one of the most common pathogens of seahorses (32, 33). This bacterial supernatant contains a large amount of harmful substances such as endotoxin (also known as LPS), which could cause cell death (34, 35). We found that both HehampII mature peptides at a minimum concentration of 12 μM could rescue seahorse embryonic cells from the death caused by pathogenic *Vibrio* supernate, respectively (**Figure 3B**), confirmed HehampII peptide’s immunological protection in seahorse. It was reported that Tilapia hepcidin could enhanced the survival rate of Chinook salmon embryo (CHSE)-214 cells infected with infectious pancreatic necrosis virus (IPNV) (36), and also, hepcidin mitigated LPS-induced inflammatory response to protect the mouse macrophage cells (37). However, much more evidences are necessary so as to receive some solid conclusion about father’s HehampII peptides functioning in protecting the incubating embryos.

### Biological Activity Detection of Seahorse HehampII Peptides

Lastly, we tested whether the proteins encoded by HehampII genes evolved new functions that adapt to the paternal care behaviour. To detect the detailed biological activity, we carried out antibacterial experiments and microbial agglutination assays on the mature peptides of *HehampII*-2 and *HehampII*-4. Interestingly, antimicrobial assays showed that neither HehampII-2 nor HehampII-4 peptides exhibited remarkable bactericidal activity against all the bacterial strains even at special higher concentrations of 96 μM (272 μg/mL of HeHampII-2, 294 μg/mL of HeHampII-4, respecitively) (**Table 1** and **Table S5**). While the encoding peptide (PC-hepc) by the hampII of large yellow croaker could kill all tested gram-positive and gram-negative bacteria at very low concentrations (**Table 1**). It has been reported that most fish hampIIs have a potent broad-spectrum antibacterial activity (16, 17, 27, 31, 38). For example, the HampII peptides from *Dicentrarchus labrax*, could significantly inhibited the growth of *Photobacterium damselae*, *Streptococcus parauberis* and *Lactococcus garviae* at a low protein concentration of 1 μM (16). The minimal effective concentrations of pufferfish (*Takifugu pardalis*) HampII peptide to all the ten strains tested were less than 40 μg/mL (17).

**Table 1:** Antimicrobial activity of seahorse HampII mature peptides.

Peptides	HehampII-2		HehampII-4		Control (PC-hepc*)	
Strains	MIC (μM)	MBC (μM)	MIC (μM)	MBC (μM)	MIC (μM)	MBC (μM)
**Gram-positive bacteria**						
*Bacillus subtilis*	—	—	—	—	1.5–3	1.5–3
*Bacillus thuringiensis*	—	—	—	—	3–6	3–6
*Micrococcus luteus*	—	—	—	—	1.5–3	1.5–3
*Staphylococcus aureus*	—	—	—	—	3–6	6–12
*Enterococcus faecalis*	—	—	—	—	3–6	3–6
Methicillin-resistant *Staphylococcus aureus (MRSA)*	—	—	—	—	3–6	3–6
Methicillin-Resistant *Staphylococcus epidermidis* (MRSE)	—	—	—	—	6–12	12–24
**Gram-negative bacterium**	—	—	—	—		
*Vibrio parahaemolyticus*	—	—	—	—	3–6	12–24
*Escherichia coli*	—	—	—	—	12–24	12–24
*Acinetobacter baumannii*	—	—	—	—	3–6	6–12
*Klebsiella pneumoniae*	—	—	—	—	1.5–3	3–6
*Salmonella typhimurium*	—	—	—	—	3–6	3–6
**Fungi**						
*Candida albicans*	—	—	—	—	—	—

**Figure 4:**
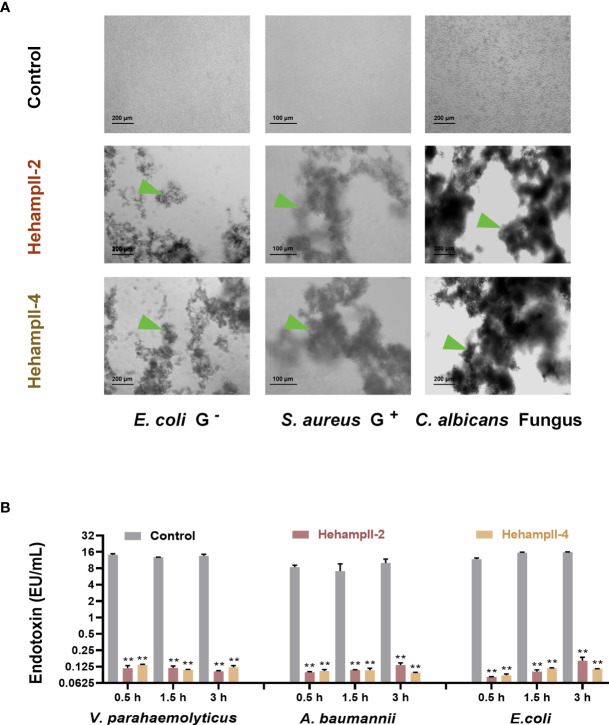
The two seahorse HampII mature peptides **(A)** showed remarkable agglutination (green triangle points to the agglutinating bacteria), and **(B)** inhibited the release of bacterial endotoxin significantly (mean ± SEM, n=3; **, indicate significant difference compared with control, *p* < 0.01).

Interestingly, we found that HehampII mature peptides possessed remarkable concentration-dependent agglutination effects on the bacteria (**Figure 4A** and **Table S5**). Both HehampII-2 and HehampII-4 mature peptides exhibit microbial agglutination to all the tested strains at a low concentration of 3 μM. Compare to the strong broad-spectrum microbial agglutination activity of HehampIIs, few reports on microbial agglutination of hampII in other teleost species were identified so far. Besides, the previous study on tiger-tail seahorse *H. comes* identified novel C-type lectins (CTLs), which were highly expressed in the brood pouch and caused erythrocyte agglutination, and were supposed to help to repress bacterial growth (13).

In addition, we measured the neutralization of the endotoxin activity of LPS from three gram-negative bacteria (*Vibrio parahaemolyticus, Escherichia coli, Acinetobacter baumannii*) by two seahorse hampII peptides. We found that both two seahorse hampII peptides performed significant neutralization ability of the endotoxin activity. The endotoxin values in the control test are almost one hundred times more than that in any hampII peptide- incubating test (**Figure 4B**). Free endotoxin released from bacteria are harmful for mammal’s pregnancy and could lead to abortion and death of embryos (39, 40). Antimicrobial proteins such as human histones H2A and H2B were highly expressed in placenta and possessing dose-dependent inhibition of the endotoxin, empower the placenta an immune-privileged site with an antimicrobial and endotoxin-neutralizing barrier against microorganisms (41). Therefore, the strong neutralization abilities of endotoxin by seahorse HehampIIs suggest their protective effect on embryonic cells from microorganisms.

The closed brood pouch of seahorse provides the ideal conditions for bacterial proliferation (filled with non-sterile seawater and the nutrient-rich pouch fluid), therefore, it is essential to balance the paternal immune molecules and commensal microbiota and maintain a pathogen-limited environment in seahorse brood pouches during pregnancy (10, 11). We supposed that these modified antibacterial functions of HampII peptides might be a kind of adaptation to reduce the lethality of embryos during pregnancy and facilitate paternal immune protection. The HehampII peptides released into the brood pouch inhabit the bacterial boom by agglutination, and also protect the embryos by avoiding the toxic effects from the free endotoxins accumulation especially in the enclosed environment of the pregnant brood pouch. In addition, one possible explanation to the loss of antibacterial activity in HehampIIs is that the antibacterial activity might also result into damage to the incubated embryos. For example, IL-37 has a strong antibacterial effect on most Gram-negative and Gram-positive bacteria, but it is also toxic to a series of host cells and tissues (42–44). Therefore, a trade-off between antibacterial function and protection of the offspring during evolution lead to the loss of the common crude sterilization function of HampIIs. Similar functional diversity of antimicrobial peptides were also found in some teolost (45). For example, HampII peptides of large yellow croaker were functionally diverse, involving in antibacterial activity, antiviral activity, and also regulation of intracellular iron metabolism. Another interesting phenomenon is that, although four hehampII genes varied a lot among each other and with the highest ratio of dN/dS, their expression pattern and function are still quite similar. In contrast, substantial variation in expression pattern and biological function were observed in line with lineage-specific gene duplication of hampIIs in other teleost species (19).

In summary, our study demonstrated that the rapid co-option of *hampII* genes, which is complemented with a weaker gene expansion but rapid variation in the seahorse ancestor, promotes the high expression of *hampII* genes in the brood pouch during male pregnancy and their function alteration. The rapid co-option reduces the major antibacterial function of the *hampII* genes, which might be an evolutionary adaptation to maintain a balance between antibacterial function and embryo-protection during male pregnancy of seahorse.

## Methods

### Fish Husbandry and Sample Preparation

All the lined seahorses (*Hippocampus erectus*) are captive-bred individuals collected from the Zhangzhou Seahorse Breeding Farm, Fujian Province, China. Seahorses were acclimated for 7 d before the experiments. Fish were maintained in seawater circulation tanks under a light-dark cycle (light: dark, 16:8 h). The salinity, light intensity, temperature, pH, and dissolved oxygen were maintained at (mean ± SD) 25 ± 1.0 ‰, 2000 ± 500 lx, 22 ± 0.5°C, 7.9 ± 0.4, and 6.5 ± 0.5 mg L^−1^, respectively. Fish were fed with frozen *Mysis* twice daily. All fish were anaesthetised with 0.05% MS222 prior to dissection. All experiments were performed in accordance with the Animal Research and Ethics Committees of the Chinese Academy of Sciences (approval number: SCSIO-IACUC-2019-000137).

### Cloning and Sequence Analysis of HampIIs

Four hepcidins identified from lined seahorse genome database (46) were cloned. All the reagents, consumables and anatomy tools were RNase free. Total RNA of seahorse liver was extracted following the instructions coming with the TRIzol Ragent (Invitrogen, Waltham, MA, USA). According to the operating steps of ReverAce qPCR RT Master Mix with gDNA Remover (Toyobo, Osaka, Japan), 1 μg of total RNA was used to synthesize first-strand cDNA. All the total RNA and cDNA were stored at -80°C. The specific PCR primers of hampIIs were used to amplify and verify the coding sequences (**Table S3**). The parameters of specific polymerase chain reaction (PCR) were as follow: denaturation at 98°C for 5 min, followed by 36 cycles at 98°C for 30 min, 60°C for 30 s, and 72°C for 30 s, and finally extension for 10 min at 72°C. The DNA product were sequenced by Sangon Biotech Co., Ltd. (Shanghai, China). Sequence alignments of HampII mature peptides were performed using MEGA.

### Phylogenetic Analysis of HampIIs

Synteny analysis of hampIIs were performed based on the genome assemblies of all the ten teleost species (**Table S1**). Search gff files showing the order of genes and fna files containing genome sequence from the project accessions or genome assemblies of target fish. We find hampIIs and their upstream and downstream genes through gff files by gene name, and confirmed the presence and order of them by BLAST (e-value = 1 × 10^−5^) based on fna files.

Phylogenetic tree was constructed according to the homologous genes of hampIIs from the published genome data including five Syngnathidae species and other five teleost species, and use the *hamp* gene from spotted gar (*Lepisosteus oculatus*) as outgroup (**Table S1**). The coding protein sequences of hampIIs were aligned using MAFFT v7.475 after removing sequences with frame-shifts or missing exons. Sites with less than five gaps were kept for phylogenetic analysis. Aligned coding protein sequences were transform back to nucleotide sequences using ‘tranalign’ in the EMBOSS toolkit. The best-fitting nucleotide and amino acid substitution model based on Akaike’s information criterion (AICc) was chosen using Mega X. The best-fit model was K80+G for nucleotide and JTT+G+I for amino acid. Bayes phylogenetic tree was constructed using MrBayes parallel 3.2.6 with 4 chains of 500,000,000 generations and 20% burn-in. Maximum likelihood phylogenetic tree was also constructed using RAxML 8.2.12 (-f a) with 1000 bootstraps. All the phylogenetic trees produced were conducted with the coding protein sequences.

The pairwise dN/dS ratios were calculated. All hampII was classified into three groups according to the species: group H: seahorses, group D: sea dragon (Syngnathidae) without seahorses, and group O: other species. The pairwise dN/dS ratios between any two hampIIs, either within group or between different groups, were calculated using PAML 4.9j (runmode = -2, model = 1, NSsites = 0). The Wilcox rank test on dN/dS values between different group pairs (like DD vs. HH) was performed using ‘wilcox.test’ in R.

### Molecular Evolution of Antimicrobial Peptides

Referring to the antimicrobial peptide database (APD3, http://aps.unmc.edu/AP/main.php) and published articles on antimicrobial peptides in fish, we focused on 25 antimicrobial peptide families: thrombin, histone, galectin, c-type lectin, collectin, ubiquitin, cyclin-dependent protein kinases (CDK-like), bovine pancreatic trypsin inhibitor (BPTI), chemokine, neuropeptide, phospholipase A2 (PLA2), transferrin, hemoglobin, defensin, actinoporin, liver-expressed antimicrobial peptide 2 (LEAP-2), beta2-microglobulin, thymosin, amyloid, enhancers of rudimentary (ERH), glyceraldehyde 3-phosphate dehydrogenase (GAPDH), saposin, hepcidin, mannose-binding lectin and lysozyme (47–51). The lined seahorse genome database was downloaded from PRJNA347499 (46), and the homologous genes of antimicrobial peptides were searched by gene names mentioned above in the annotation file, and ascertained by BLAST (e-value = 1 × 10^−5^) based on amino acid sequences in cds file. Maximum likelihood phylogenetic tree (species involved are listed in **Table S1**) was constructed by RAxML 8.2.12 (-f a) using Jones’ JTT+G amino acid substitution model with 1000 bootstraps. The dN/dS ratios of all branches, include the seahorse ancestor branch, were calculated under the free-ratios model (model=1, NSsites=0) of PAML 4.9j. The estimated dN/dS of the branch leading to the seahorse ancestor were fetched for comparison.

### Gene Expression Patterns of HehampII Family During Seahorse’s Breeding Cycle

In order to collect the placenta tissue of brood pouches of seahorses at different pregnancy stages, reproductively mature non-pregnancy male and female lined seahorses derived from breeding farm were maintained together (males = 60; female = 60; body length 12-15 cm, wet weight 3-6 g) in the seawater circulation system, and all fish were allowed to mate freely. The male pregnant seahorses were sampled at certain stage, then anesthetized and dissected to collect the brood pouch placenta, liver, and offspring (or embryos). The brood pouch inner layers from the non-pregnant but courtship-active male seahorses were defined as pre-pregnancy stage (*n* = 8). The pregnancy seahorses were sampled at three sub-stages according to the number of day post mating: Early Pregnancy (less than 4 d; *n* = 7), Middle Pregnancy (approximately 4–10 d; *n* = 8), and Late Pregnancy (approximately 11–15 d; *n* = 8). And the males at one day post-delivery were termed to be in the Post-Pregnancy period (52). In addition, the livers from seahorse were sampled too, it should be mentioned that the livers from the early, middle and late pregnancy seahorse serves as a mixed sample. All the collected tissues were surgically sampled and immediately frozen in liquid nitrogen and stored at -80°C for further analysis.

### Real-Time Quantitative PCR

The expression levels of seahorse hampIIs were detected using quantitative real-time PCR (qPCR). All specific primers for qPCR are listed in **Table S3**. According to the instructions provided with the SYBR Green I kit (Toyobo, Japan), qPCR was conducted on a Roche Light-Cycler 480 real-time PCR system. The qPCR amplification system was listed in **Table S6**. The primer efficiency of all the HehampIIs was measured using the standard curves generated by a series of plasmid constructs (**Figure S5**). *β*-actin was used as the reference genes. The qPCR cycling parameters were as follows: 95°C for 3 min, followed by 40 cycles at 95°C for 30 s, 60°C for 30 s, and 72°C for 20 s. The melting curve was checked to exclude nonspecific amplification products (**Figure S6**). Gene expression levels were determined using the threshold cycle (Ct) values converted using fluorescence data.

### Expression of Seahorse HampII Genes in Response to Lipopolysaccharide Stimulation *In Vivo*

The sidewall tissue of the brood pouch of male seahorses (12.7 ± 0.45 g) was injected with 100 μL of either LPS (2 μg/μL, Sigma Aldrich, *Escherichia coli* O111: B4) in phosphate-buffered saline (PBS), or with only sterile PBS in the sham control. Seahorses were collected at each sampling time point, including at 6, 12, and 24 h post injection (hpi) (*n* = 5 each group). The liver and placenta tissue of the brood pouch were surgically sampled and immediately frozen in liquid nitrogen and stored at -80°C for further analysis.

### Cytotoxicity Test *In Vitro*

Two HampII mature peptides of lined seahorse (HehampII-2 and HehampII-4), of which the amino acid sequences, molecular mass, and purity are summarised in **Table S4**, were synthesised by GenScript Biotech (Nanjing, China) using the solid-phase peptide synthesis method.

The seahorse embryo cell line established previously in our lab were used for cytotoxicity test *in vitro* (53). The cells were cultivated in the Dulbecco’s modified eagle medium (DMEM): Ham’s nutrient mixture F-12 (1:1) medium (DMEM/F12) supplemented with antibiotics (penicillin, 400 U/mL; streptomycin, 400 µg/mL; Gibco BRL) and fetal bovine serum (FBS, 20%). Embryo cells were transferred into 6-well cell culture plates containing 6 ml DMEM per well. *V. parahaemolyticus* was centrifuged at 4,000 × *g* after cultured for 12 h, and the supernate was filtered with a 0.22 μm filter. During experiment, several reagents were involved: DMEM (without antibiotics and FBS), PBS (containing 1‰ DMSO), *Vibrio* supernate, HehampIIs (192 μM; dissolved in PBS with 1‰ DMSO). And several groups were set up this experiment: normal group (5.25 mL DMEM + 0.75 mL PBS), sham control group (4.95 mL DMEM + 0.375 mL PBS + 0.375 mL HehampIIs + 0.3 mL *Vibrio* supernate), two 12 μM HehampIIs groups (5.325 mL DMEM + 0.375 mL HehampIIs + 0.3 mL *Vibrio* supernate) and two 24 μM HehampIIs groups (4.95 mL DMEM + 0.75 mL HehampIIs + 0.3 mL *Vibrio* supernate). The cell viability was detected using Cell Counting Kit-8 (MCE, USA) after 12 h-incubation.

### Biological Activity Detection of Seahorse HampII Mature Peptides

#### Antimicrobial Assay

Antibacterial assay was performed following the liquid growth inhibition assay protocol reported previously (54). And the synthetic mature peptide from the large yellow croaker (*Pseudosciaena crocea*) HampII (PC-hepc) was also synthesised and used as a reference in this study (**Table S4**) (38). The purity of all three synthetic polypeptides was over 95%, making the biological activity detection assay possible. These peptides were stored in dry powder form at -80°C before use. To evaluate the antibacterial activity of these synthetic peptides, a variety of fish pathogenic strains (*Acinetobacter baumannii*, *Bacillus subtilis*, *Bacillus thuringiensis*, *Candida albicans*, *Escherichia coli*, *Enterococcus faecalis*, *Klebsiella pneumoniae*, *Micrococcus luteus*, methicillin-resistant *Staphylococcus aureus* (MRSA), methicillin-Resistant *Staphylococcus epidermidis* (MRSE), *Staphylococcus aureus*, *Salmonella typhimurium*, and *Vibrio parahaemolyticus*) were cultured and tested in this study. These bacteria, marine bacteria, or fungi were cultivated in nutrient broth (NB), Mueller Hinton broth (MH broth), 2216E and YPD medium, respectively. Microbial cells were collected by centrifugation at 4000 × *g* at room temperature for 5 min when cultured to the logarithmic phase, and then resuspended in the corresponding medium. Bacteria (10^4^ CFU/well) and fungi (10^3^ CFU/well) were cultured using a serial dilution of polypeptides in a 96-well microbe culture plate at their optimum temperature for 48 h. HehampII peptides were dissolved in sterile water with 1‰ DMSO to obtain final protein concentrations of 96, 48, 24, 12, 6, 3, and 1.5 μM by serial dilution with sterilised water. Minimum inhibitory concentration (MIC) is defined as the lowest protein concentration that can inhibit the visible growth of microorganisms. Based on the MIC, the mixture with no visible growth was inoculated into the corresponding agar plates and cultured at an optimal temperature for 48 h. The minimum bactericidal concentration (MBC) is defined as the concentration of protein that prevents microbial growth of more than 99.9%. All assays were performed in triplicate.

#### Microbial Agglutination Assay

Three typical strains (gram-negative bacterium: *E. coli*, Gram-positive bacterium: *S. aureus*, Fungus: *C. albicans*) were used to test the microbial agglutination of seahorse HampII peptides. The strains were centrifuged at 4000 × *g* for 5 min to collect the bacterial cells, and then inoculated into the corresponding medium and cultured for 12–18 h. The strains were washed three times with TBS buffer (10 mM Tris-HCl, 150 mM NaCl, pH 7.4) and then resuspended in TBS buffer to ensure that the OD_600_ reached 0.6. In the agglutination experiment, 10 mM CaCl_2_ was added, and the final OD_600_ value of the bacterial solution was 0.3 by mixing with the peptides. HehampIIs dissolved in 1‰ DMSO were diluted with sterilised water to a final concentration of 96, 48, 24, 12, 6, and 3 μM. Bovine serum albumin (BSA) (2 mg mL^−1^) with 1‰ DMSO was used as the control. The agglutination reactions were observed using an optical microscope. The free bacterial cells were sucked out, diluted, and then coated on agar plate with corresponding media, and the CFU were calculated after incubated at optimum temperature for 24-48 h. The percentage of CFU of free bacterial cells in the test to that in the control was evaluated, so the degree of microbial agglutination was measured. All assays were performed in triplicate.

#### Endotoxin Neutralization Test

Three gram-negative bacteria (*A. baumannii, E. coli*, and *V. parahaemolyticus*) were used to test the neutralization ability of two seahorse HehampII peptides, respectively. The pellets of strains were collected after overnight incubation at the optimal temperature, washed three times with PBS, and resuspended to 10^5^ CFU mL^−1^ with PBS. The bacterial suspensions were mixed with HeHampII-2 peptide and HeHampII-4 peptide (at a final concentration of 6 μM), respectively, and the sterile water with 1‰ DMSO were used as a control. After incubation for 0.5, 1.5, and 3 h at the optimum temperature, the mixture was filtered with a 0.22 μm filter, and the endotoxin value was determined according to the instructions of the Bioendo KC Endotoxin Test Kit (Kinetic Chromogenic Assay) (BIOENDO, China). All assays were performed in triplicate. All reagents and consumables used in this experiment were sterile and free of endotoxins.

### Statistical Analysis

All data were analysed using GraphPad 6 software and presented as the mean ± standard error of the mean (SEM). Unpaired Student’s *t*-tests or one-way ANOVAs followed by Tukey’s tests were used to analyse the statistical differences, and differences were considered statistically significant at *p <* 0.05.

## Data Availability Statement

The datasets presented in this study can be found in online repositories. The names of the repository/repositories and accession number(s) can be found in the article/**Supplementary Material**.

## Ethics Statement

The animal study was reviewed and approved by Animal Research and Ethics Committees of the Chinese Academy of Sciences (approval number: SCSIO-IACUC-2019-000137).

## Author Contributions

QL and GQ supervised the project and designed the research. ZC & WX. performed the genome and genetic analyses; WX, YW, HZ, HJ, and YZ. performed qPCR & biological activity detection; WX, GQ, ZC and MQ performed original draft writing and drawing figures; QL & YZ reviewed the writing. All authors contributed to the article and approved the submitted version.

## Funding

This research was supported by the Key Special Project for Introduced Talents Team of Southern Marine Science and Engineering Guangdong Laboratory (Guangzhou) (GML2019ZD0407 and GML2019ZD0401), the National Natural Science Foundation of China (42076131, 42176120), the Strategic Priority Research Program of the Chinese Academy of Sciences (XDB42030204), the Hainan Province Science and Technology Special Fund (ZDYF2021SHFZ109), the Key Deployment Project of Centre for Ocean Mega-Research of Science, Chinese Academy of Sciences (COMS2020Q14), and the Hainan Provincial Science and Technology Plan Sanya Yazhou Bay Science and Technology City Joint Project (320LH046).

## Conflict of Interest

The authors declare that the research was conducted in the absence of any commercial or financial relationships that could be construed as a potential conflict of interest.

## Publisher’s Note

All claims expressed in this article are solely those of the authors and do not necessarily represent those of their affiliated organizations, or those of the publisher, the editors and the reviewers. Any product that may be evaluated in this article, or claim that may be made by its manufacturer, is not guaranteed or endorsed by the publisher.

## Supplementary Material

Original datasets to this article can be found in the electronic Supplementary files (https://doi.org/10.6084/m9.figshare.19404554.v1) (55)
