# Supplementary material for: Hepcidin Gene Co-Option Balancing Paternal Immune Protection and Male Pregnancy
Source: Front Immunol. 2022 Apr 22;13:884417. doi: 10.3389/fimmu.2022.884417 (PMC9073008; doi:10.3389/fimmu.2022.884417)
Supplement: Supplementary file 1 [file DataSheet_1.docx]

**Supplementary data**

**Hepcidin gene co-option balancing paternal immune protection and male pregnancy**

Wanghong Xiao ^a,b,c,#^, Zelin Chen ^a,#^, Yanhong Zhang ^a,b^, Yongli Wu ^a^, Han Jiang ^a^, Huixian Zhang ^a^, Meng Qu ^a^, Qiang Lin ^a,b,c^*, Geng Qin ^a,b^*

^a^ *Southern Marine Science and Engineering Guangdong Laboratory (Guangzhou), Guangzhou 511458, PR China*

^b^ *CAS Key Laboratory of Tropical Marine Bio-resources and Ecology, South China Sea Institute of Oceanology, Chinese Academy of Sciences, Guangzhou 510301, PR China*

^c^ *University of the Chinese Academy of Sciences, Beijing 100049, PR China*

* Corresponding authors.

*E-mail address*: qingeng@scsio.ac.cn (G. Qin); linqiang@scsio.ac.cn (Q. Lin)

* Corresponding authors.

*E-mail address*: qingeng@scsio.ac.cn (G. Qin); chzelin@scsio.ac.cn (Z. Chen); linqiang@scsio.ac.cn (Q. Lin)

## Content:

**1 Figures S1–4**

**2 Tables S1–5**

**3 Informatin for qPCR experiments (Figure S5-6, Table S6)**

## 1 Figures S1–4


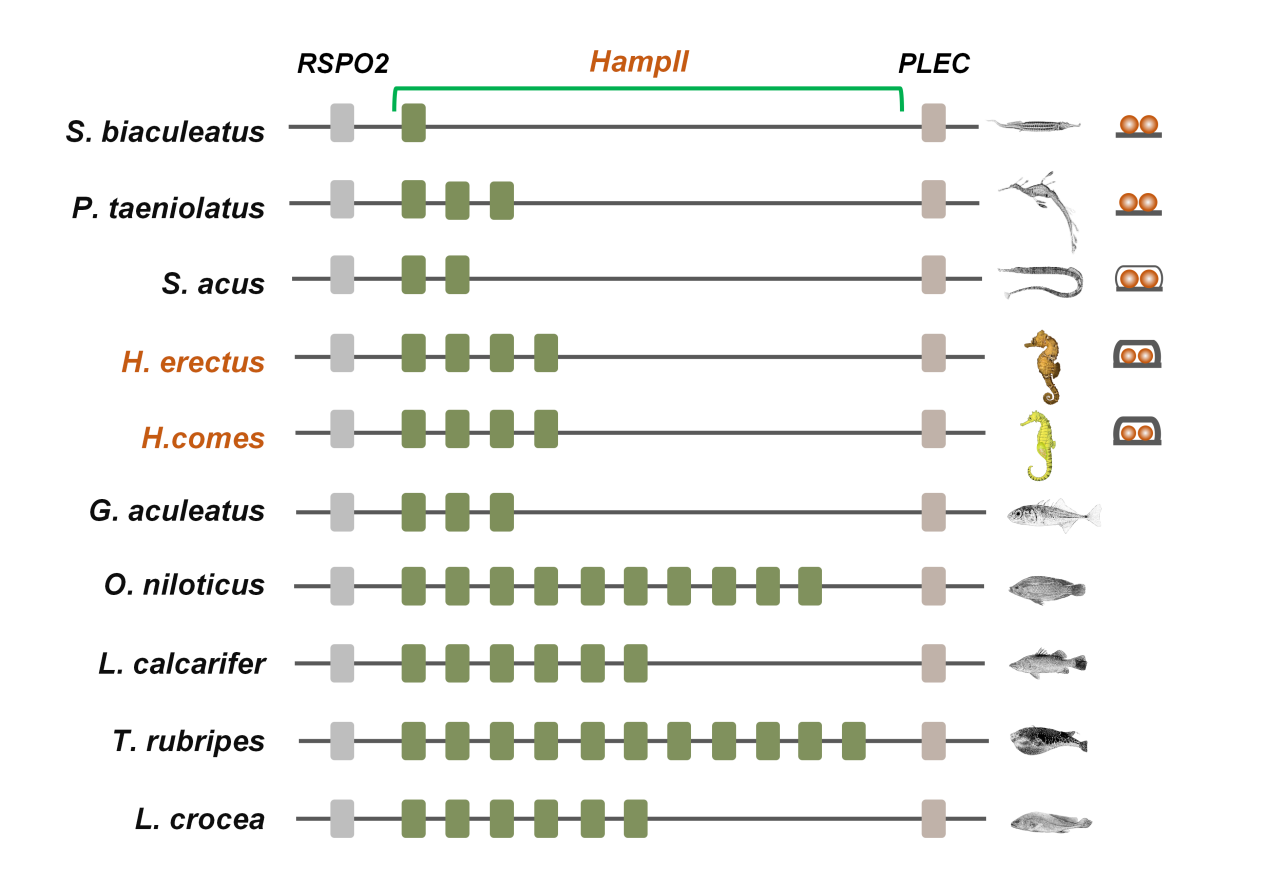


### Figure S1. Synteny analysis showed that the hampII gene family had multiple tandem duplicates. HampIIs were denoted in green, and brood pouch type were illustrated on the right.


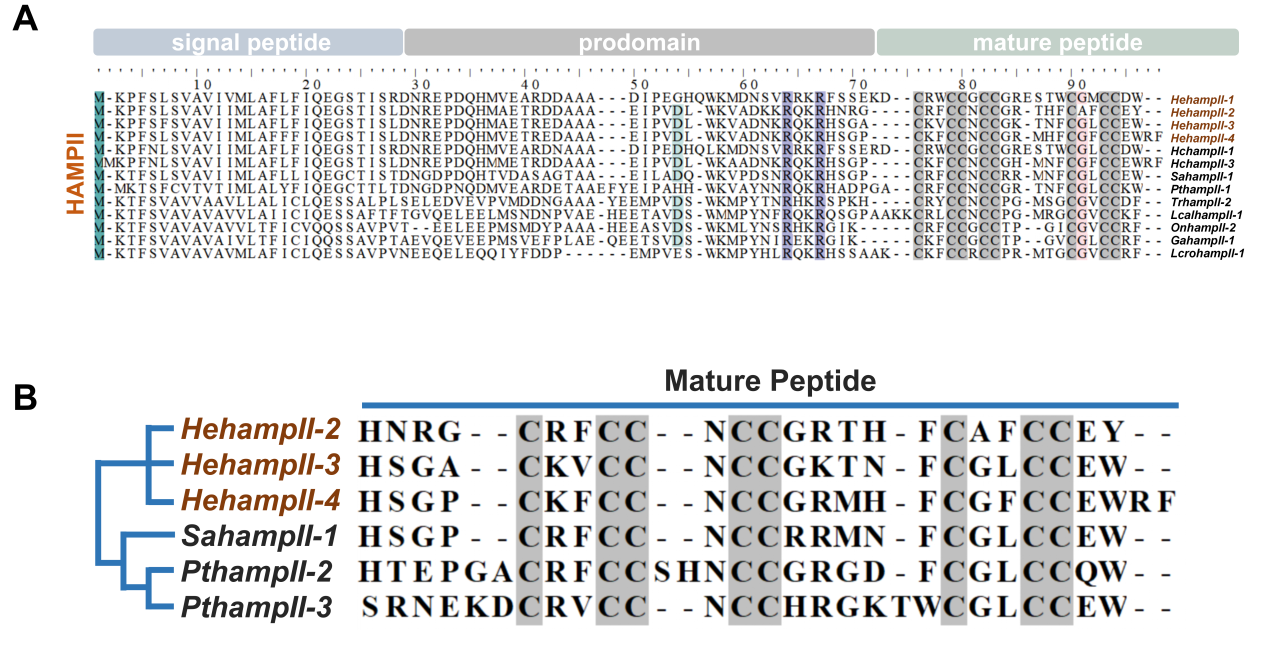


### Figure S2. Sequence alignment of hampII genes. The comparative sequence analysis showed that seahorse hampII genes varied remarkably from other teleost species (A). Lined seahorse *HampII* (*HehampII*) coding proteins vary considerably in the mature peptide region among different copies and with comparison to their relative the greater pipefish (*Syngnathus acus*) (*SahampII*) and common seadragon (*Phyllopteryx taeniolatus*) (*PthampII*) (B).


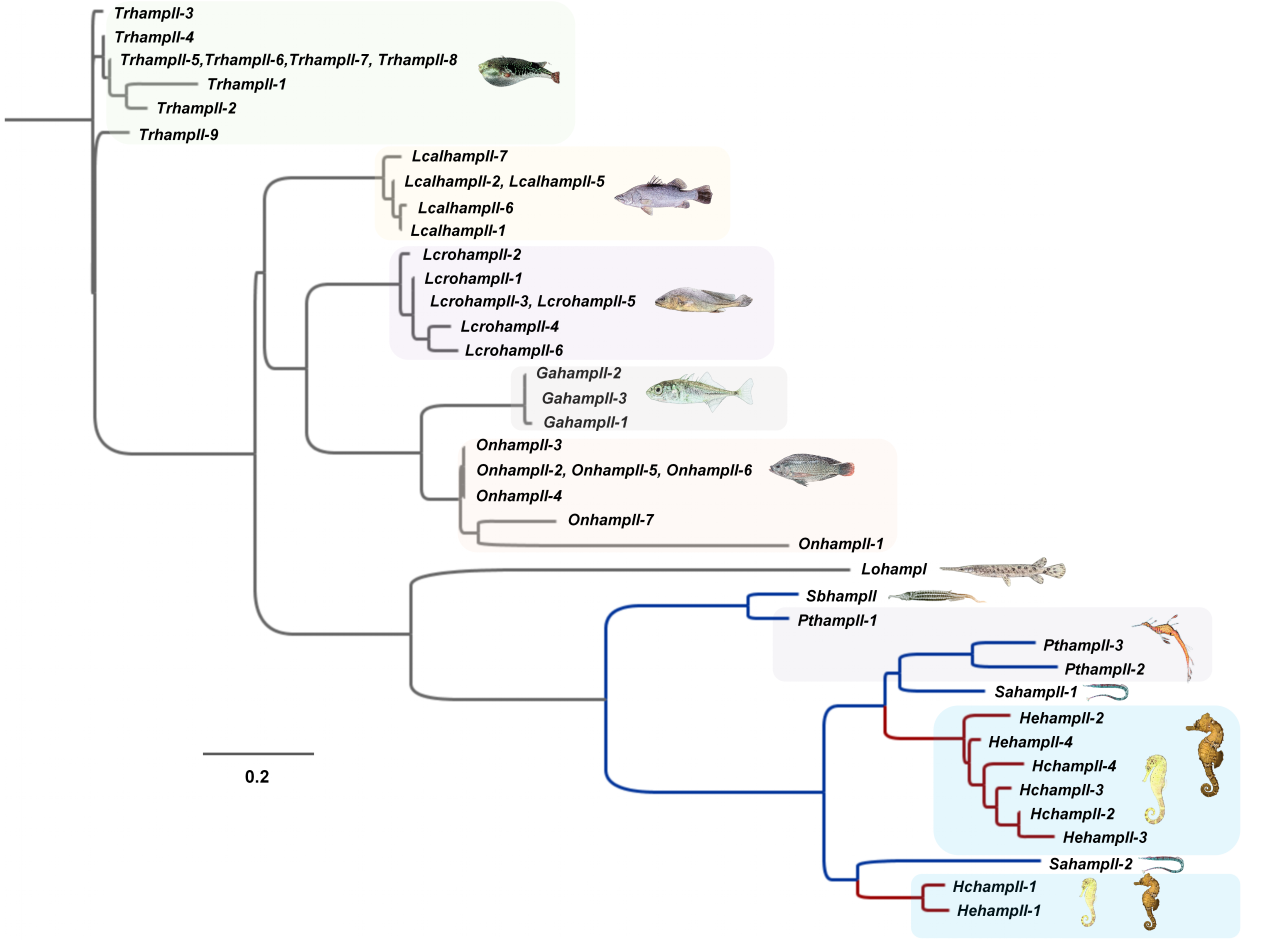


### Figure S3. Maximum likelihood phylogenetic tree showed that evolutionary rates of Syngnathidae hampIIs were faster than that of other teleosts. HampIIs in Syngnathidae were highlighted in light blue background, seahorse hampIIs in red line, and other Syngnathidae hampIIs in blue line.


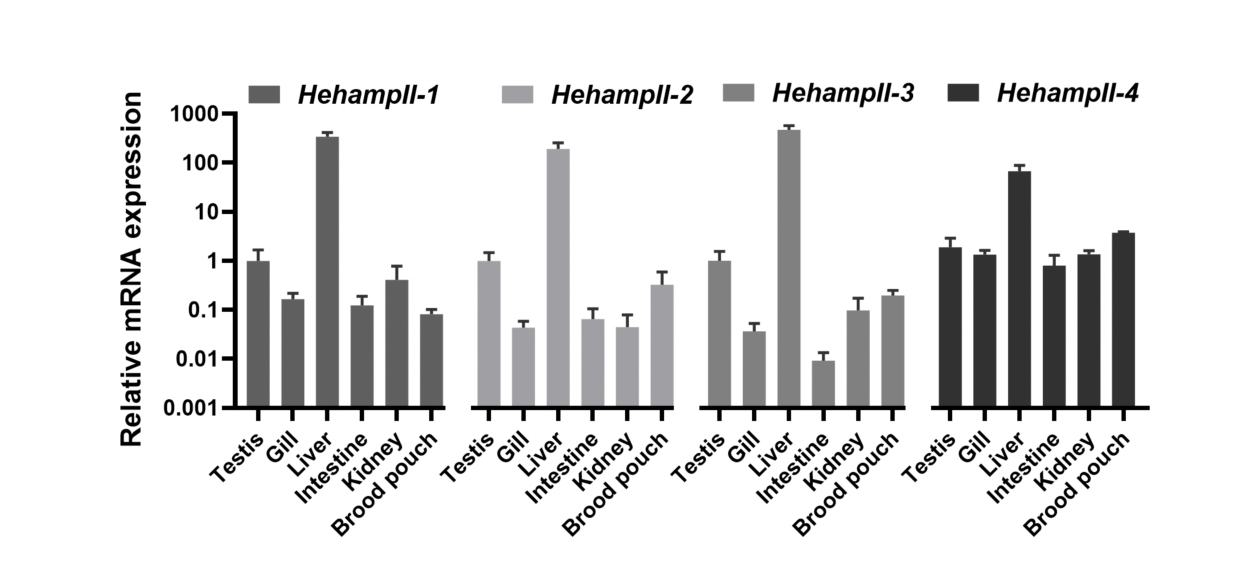


### Figure S4. Seahorse *HehampIIs* expression were detected in most tissues, and the highest expression among all *HehampIIs* was detected in the liver (n ≥ 5, mean ± SEM).

###

## 2 Tables S1–6

### Table S1. Gene IDs of hampII sequences used for phylogenetic trees and synteny analysis

| Species | BioProject or Assembly | Gene name and IDs | Location | Begin-End | CDS | Pep |
| --- | --- | --- | --- | --- | --- | --- |
| *Hippocampus erectus* | PRJNA347499 | HehampII-1: EVM0018308  (GeneBank: OK144241) | Chr3, Ctg28 | 1609753-1610959 | >HehampII-I EVM0018308.1 ATGAAGCCCTTCAGTTTGTCTGTTGCAGTGATCGTCATGCTTGCCTTCCTTTTTATCCAGGAGGGTTCTA  CCATCTCTCGTGATAATCGGGAGCCTGACCAGCATATGGTGGAAGCAAGAGATGACGCAGCTGCTGACAT  ACCGGAAGGCCACCAGTGGAAGATGGACAATAGCGTCAGACGCAAACGCTTCAGCAGTGAAAAGGATTGC  CGCTGGTGCTGCGGTTGCTGTGGTCGAGAATCGACATGGTGCGGTATGTGCTGTGACTGGTGA | >HehampII-I EVM0018308.1 MKPFSLSVAVIVMLAFLFIQEGSTISRDNREPDQHMVEARDDAAADIPEGHQWKMDNSVRRKRFSSEKDCRWCCGCCGRESTWCGMCCDW |
|  |  | HehampII-2: EVM0015696  (GeneBank: OK144242) |  | 1619076-1619500 | >HehampII-2 EVM0015696.1  ATGAAGCCCTTCAGTTTGTCTGTTGCAGTGATCATCATGCTTGCCTTCCTTTTTATTCAGGAGGGTTCCA  CCATCTCTCTTGATAATAGGGAGCCTGACCAGCATATGGCGGAAACAAGAGATGACGCAGCTGCTGAGAT  CCCAGTGGACTTATGGAAGGTGGCAGACAAAAAGAGGCAGAAACGTCATAATAGAGGTTGCAGATTTTGC  TGCAACTGCTGCGGTCGTACGCATTTCTGTGCCTTTTGCTGCGAATATTGA | >HehampII-2 EVM0015696.1  MKPFSLSVAVIIMLAFLFIQEGSTISLDNREPDQHMAETRDDAAAEIPVDLWKVADKKRQKRHNRGCRFC  CNCCGRTHFCAFCCEY |
|  |  | HehampII-3: EVM0017248  (GeneBank: OK144243) |  | 1621976-1622632 | >HehampII-3 EVM0017248.1  ATGAAGCCCTTCAGTTTTTCTGTTGCAGTGATCATCATGCTTGCCTTCCTTTTTATTCAGGAGGGTTCCA  CCATCTCTCTTGATAATAGGGAGCCTGACCAGCATATGGCGGAAACAAGAGAAGACGCAGCTGCTGAGAT  CCCAGTGGACTTATGGAAGGTGGCAGACAACAAGAGGCAGAAACGCCATAGTGGAGCTTGCAAAGTTTGC  TGCAACTGCTGCGGTAAAACAAATTTCTGTGGCTTGTGCTGCGAATGGTGA | >HehampII-3 EVM0017248.1  MKPFSFSVAVIIMLAFLFIQEGSTISLDNREPDQHMAETREDAAAEIPVDLWKVADNKRQKRHSGACKVCCNCCGKTNFCGLCCEW |
|  |  | HehampII-4: EVM0003101  (GeneBank: OK144244) |  | 1625688-1626117 | >HehampII-4 EVM0003101.1ATGAAGCCCTTCAGTTTGTCTGTTGCAGTGATCATCATGCTTGCCTTCTTTTTTATTCAGGAGGGTTCCA  CCATCTCTCTTGATAATAGGGAGCCTGACCAGCATATGGTGGAAACAAGAGAAGACGCAGCTGCTGAGAT  CCCAGTGGACTTATGGAAGGTGGCAGACAACAAGAGGCAGAAACGCCATAGTGGACCTTGCAAATTTTGCTGCAACTGCTGCGGTCGTATGCATTTCTGTGGCTTCTGCTGCGAATGGAGATTCTAA | >HehampII-4 EVM0003101.1 MKPFSLSVAVIIMLAFFFIQEGSTISLDNREPDQHMVETREDAAAEIPVDLWKVADNKRQKRHSGPCKFC  CNCCGRMHFCGFCCEWRF |
| *Hippocampus comes* | GCA_001891065.1 | HchampII-1: XM_019892993.1 | NW_017806059.1 | 632368-633774 | >HchampII-1 XM_019892993.1  ATGAAGCCCTTCAATTTGTCTGTTGCAGTGATCATCATGCTTGCCTTCCTTTTTATCCAGGAGGGTTCTACCATCTCTCGTGATAATCGGGAGCCTGACCAACATATGGTGGAAGCAAGAGATAACGCAGCTGCTGACATCCCGGAAGACCACCAGTTGAAGATGGACAATAGCGTCAGACGCAAACGCTTCAGCAGTGAAAGGGATTGCCGCTGGTGCTGCGGTTGCTGTGGTCGAGAATCGACATGGTGTGGTCTGTGCTGTGACTGGTGA | >HchampII-1 XM_019892993.1  MKPFNLSVAVIIMLAFLFIQEGSTISRDNREPDQHMVEARDNAAADIPEDHQLKMDNSVRRKRFSSERDCRWCCGCCGRESTWCGLCCDW |
|  |  | HchampII-2: XM_019892989.1 |  | 636233-636938 | >HchampII-2 XM_019892989.1  ATGATGAAGCCCTTCAATTTGTCTGTTGCAGTGATCATCATGCTTGCCTTCCTTTTTATTCAGGAGGGTTCCACCATCTCTCTTGACAATAGGGAGCCTGACCAGCATATGATGGAAACAAGAGATGACGCAGCTGCTGAGATCCCAGTGGACTTATGGAAGGTGGCAGACAACAAGAGGCAGAAACGTCATAATGGAGCTTGCAAAGTTTGCTGCAACTGCTGCGGTCAAATAAATTTCTGTGGCTTGTGCTGTGAATGGTGA | >HchampII-2 XM_019892989.1  MMKPFNLSVAVIIMLAFLFIQEGSTISLDNREPDQHMMETRDDAAAEIPVDLWKVADNKRQKRHNGACKVCCNCCGQINFCGLCCEW |
|  |  | HchampII-3: XM_019892988.1 |  | 639730-640358 | >HchampII-3 XM_019892988.1  ATGATGAAGCCCTTCAATTTGTCTGTTGCAGTGATCATCATGCTTGCCTTCCTTTTTATTCAGGAGGGTTCCACCATCTCTCTTGACAATAGGGAGCCTGACCAGCATATGATGGAAACAAGAGATGACGCAGCTGCTGAGATCCCAGTGGACTTATGGAAGGCGGCAGACAACAAGAGGCAGAAACGCCATAGTGGACCTTGCAAATTTTGCTGCAACTGCTGCGGTCATATGAATTTCTGTGGCTTTTGCTGCGAATGGAGATTCTAA | >HchampII-3 XM_019892988.1  MMKPFNLSVAVIIMLAFLFIQEGSTISLDNREPDQHMMETRDDA  AAEIPVDLWKAADNKRQKRHSGPCKFCCNCCGHMNFCGFCCEWRF |
|  |  | HchampII-4: XM_019892990.1 |  | 643116-643800 | >HchampII-4 XM_019892990.1  ATGATGAAGCCCTTCAATTTGTCTGTTGCAGTGATCATCATGCTTGCCTTCCTTTTAATTCAGGAGGGTTGCACCATCTCTATTGACAATTGGGAGCCTGACCAGCATATGATGGAAACAAGAGATGACGCAGCTGCTGAGATCCCAGTGGACTTATGGAAGGTGGCAGACAACAAGAGGCAGAAACGTCATAATGGAGCTTGCAAATTTTGCTGCAACTGCTGCGGTCGTACGCATTTCTGTGCCTTTTGCTGCGAATGGTGA | >HchampII-4 XM_019892990.1  MMKPFNLSVAVIIMLAFLLIQEGCTISIDNWEPDQHMMETRDDA  AAEIPVDLWKVADNKRQKRHNGACKFCCNCCGRTHFCAFCCEW |
| *Syngnathus acus* | GCA_901709675.2 | SahampII-1: XM_037273744.1 | NC_051101.1 | 5046311-5051526 | >SahampII-1 XM_037273744.1  ATGAAGACCTTCAGTTTGTCTGTTGCAGTGATCATCATGCTTGCTTTCCTTCTGATTCAGGAGGGTTGCACCATCTCTACTGATAATGGGGATCCTGACCAGCATACAGTGGACGCAAGTGCAGGAACTGCTGCTGAGATACTGGCAGACCAATGGAAGGTGCCAGACAGCAACAGGCAGAAACGCCACAGTGGACCTTGCCGCTTTTGCTGTAATTGCTGTCGTCGTATGAATTTCTGTGGCCTCTGCTGCGAATGG | >SahampII-1 XM_037273744.1  MKTFSLSVAVIIMLAFLLIQEGCTISTDNGDPDQHTVDASAGTAAEILADQWKVPDSNRQKRHSGPCRFCCNCCRRMNFCGLCCEW |
|  |  | SahampII-2: XM_037273744.1 |  | 5046311-5051526 | >SahampII-2 XM_037273744.1  ATGAAGGTCTTCAGTGTGATCATCATGCTTGCTTTCCTTTTTACTCAGGAGGGTTGTACCTTCTCTACTAATAATGGGGATCCCAACCAGCATTTGATGAACGCAAGAGCTGAAGCAGCTGCCGAGATCCACGCGGACCAATGGAAGGAAGAGGATTTGGTAAGAGCGAGAGATGAAGTTTTGGAAAAATTTGAGATGCAATCAGATGAACCAAAGATGGATTATGGTGCCAGACCTGTCCGCAATGCGAGAGATTGCCGCTGGTGCTGTGGCTGCTGTGGTAAAGAACCTACATGGTGTGGTCTCTGCTGTGACTGG | >SahampII-2 XM_037273744.1  MKVFSVIIMLAFLFTQEGCTFSTNNGDPNQHLMNARAEAAAEIHADQWKEEDLVRARDEVLEKFEMQSDEPKMDYGARPVRNARDCRWCCGCCGKEPTWCGLCCDW |
| *Phyllopteryx taeniolatus* | PRJNA669508 | PthampII-1: EVM0012287.1  (GeneBank: OK144246) | Lachesis_group5 | 14698657-14699272 | >PthampII-1 EVM0012287.1  ATGATGAAGACCAGTTTTTGTGTCACAGTGACGATCATGCTTGCCCTCTATTTTATTCAGGAGGGTTGCACCACCTTGACTGATAATGGGGATCCTAACCAGGATATGGTGGAAGCAAGAGATGAAACAGCTGCTGAATTCTATGAGATCCCAGCGCACCACTGGAAGGTGGCATACAACAACAGGCAGAAACGCCACGCTGACCCTGGAGCATGCCGCTTTTGCTGTAACTGCTGCGGTCGTACGAATTTCTGCGGCCTCTGCTGTAAATGG | >PthampII-1 EVM0012287.1  MMKTSFCVTVTIMLALYFIQEGCTTLTDNGDPNQDMVEARDETAAEFYEIPAHHWKVAYNNRQKRHADPGACRFCCNCCGRTNFCGLCCKW |
|  |  | PthampII-2: EVM0013394.1  (GeneBank: OK144247） |  | 14693246-14693873 | >PthampII-2 EVM0013394.1  ATGAAGACCTTCAGGTTTTCTGTTGCAGTAATGATCATACTTGCCCTCTTTTATATTCAAGATGGTTACACCTTATCTACTGATAATGGGGATCCTAACCAGGATATGGTGAAAGCAAGGGATGAAGAGATGATCTATGAGATCCCAGCGGACCACTGGAAGGTGGCATCCAACAACAGACAGAAACGTCACACTGAACCTGGAGCTTGCCGCTTTTGCTGTTCGCATAACTGCTGTGGCCGGGGGGATTTCTGCGGCCTCTGCTGTCAATGG | >PthampII-2 EVM0013394.1  MKTFRFSVAVMIILALFYIQDGYTLSTDNGDPNQDMVKARDEEMIYEIPADHWKVASNNRQKRHTEPGACRFCCSHNCCGRGDFCGLCCQW |
|  |  | PthampII-3: EVM0002019.1  (GeneBank: OK144248） |  | 14683885-14683971 | >PthampII-3 EVM0002019.1  ATGAAGACATTCAAGATTGCAGTTGCAGTCATCCTTTTGCTCACCTATATTTTAATACAGGAGAGCTGTGCTTCGCCATTTACTGAAGAAGAAGTAGAAGTGAGAGATGATGAAGTTTTGGAACATTTTGAGATGCAATCCGAGCAATCAAAGATGGGTGATCATGTCAGAGATAAACGCAGCAGGAATGAGAAAGATTGCCGCGTATGCTGTAATTGCTGTCATCGTGGGAAAACATGGTGTGGTCTCTGCTGTGAATGG | >PthampII-3 EVM0002019.1  MKTFKIAVAVILLLTYILIQESCASPFTEEEVEVRDDEVLEHFEMQSEQSKMGDHVRDKRSRNEKDCRVCCNCCHRGKTWCGLCCEW |
| *Syngnathoides biaculeatus* | PRJNA669508 | Sbhamp-II: EVM0003541.1  (GeneBank: OK144245） | LG05 | 21575068-21575683 | >SbhampII EVM0003541.1  ATGAAGGTATTCAACATTGCGGTTGCAGTTATCATTTTGCTCAGCTATATTTTATTACAAGAGAGCTCTG  CCTCGCCATTTACTGAAGAAAAAGTTGAAGTGAGAGACGACAATTTGGAAGCTTTTGAGATGCAGTCAGA  GCCATCAAAGATGAATGATAATGTCAGAGACAAACGAAGCATGGATGAAAAAG | >SbhampII EVM0003541.1 MKVFNIAVAVIILLSYILLQESSASPFTEEKVEVRDDNLEAFEMQSEPSKMNDNVRDKRSMDEKDCRVCC  NCCHRGPTWCGLCCEW |
| *Takifugu rubripes* | GCA_901000725.2 | TrhampII-1: XM_029838813.1, | NC_042291.1 | 8140113-8143120 | >TrhampII-1 XM_029838813.1  ATGAAGACCTTCAGTGTTGCTGTTGTTGCGGCCGTCCTGCTGGCCCTCATTTGTCTCCAGGAGAGCTCTGCTCTTCCTCTCAGTGAATTGGAAGATGTGGAGGTGCCAGTGATGGATGATAATGGAGCTGCTGTATATGAAGAGATGCCAGTGGACTCCTGGAAGATGCCGTATACCCACATGCGCCATCATCGTCCTGCACGCTGCAGGTTTTGCTGTCGATGCTGTCCTGGAATGCAGGGATGTGGAATCTGTTGCGATTTCTAA | TrhampII-1 XM_029838813.1  MKTFSVAVVAAVLLALICLQESSALPLSELEDVEVPVMDDNGAAVYEEMPVDSWKMPYTHMRHHRPARCRFCCRCCPGMQGCGICCDF |
|  |  | TrhampII-2: XM_029838811.1, |  | 8146817-8147614 | >TrhampII-2 XM_029838811.1  ATGAAGACCTTCAGTGTTGCTGTTGTTGCGGCCGTCCTGCTGGCCCTCATTTGTCTCCAGGAGAGCTCTGCTCTTCCTCTCAGTGAATTGGAAGATGTGGAGGTGCCAGTGATGGATGATAATGGAGCTGCTGCATATGAAGAGATGCCAGTGGACTCCTGGAAGATGCCGTATACCAACAGACACAAGCGGAGTCCTAAACACTGCAGGTATTGCTGTAATTGCTGTCCTGGAATGAGTGGATGTGGTGTCTGCTGTGACTTCTGA | >TrhampII-2 XM_029838811.1  MKTFSVAVVAAVLLALICLQESSALPLSELEDVEVPVMDDNGAAAYEEMPVDSWKMPYTNRHKRSPKHCRYCCNCCPGMSGCGVCCDF |
|  |  | TrhampII-3: XM_029838816.1, |  | 8148985-8149782 | >TrhampII-3 XM_029838816.1  ATGAAGACCTTCAGTGTTGCTGTTGTTGCGGCCGTCCTGCTGGCCCTCATTTGTCTCCAGGAGAGCTCTGCTCTTCCTCTCAGTGAAGTGGACGATGTGGAGGTGCCAGTGATGGATGATAATGGAGCTGCTGTATATGAAGAGATGCCAGTGGACTCCTGGAAGATGCCGTATACCAACAGACACAAGCGGAGTCCTAAACGCTGCAAGTTTTGCTGTAATTGCTGTCCTAGAATGAGTGGATGTGGTGTCTGCTGCAGGTTCTGA | >TrhampII-3 XM_029838816.1  MKTFSVAVVAAVLLALICLQESSALPLSEVDDVEVPVMDDNGAAVYEEMPVDSWKMPYTNRHKRSPKRCKFCCNCCPRMSGCGVCCRF |
|  |  | TrhampII-4: XM_029838810.1, |  | 8151166-8151965 | >TrhampII-4 XM_029838810.1  ATGAAGACCTTCAGTGTTGCTGTTGTTGCGGCCGTCCTGCTGGCCCTCATTTGTCTCCAGGAGAGCTCTGCTCTTCCTCTCAGTGAAGTGGAAGATGTGGAGGTGCCAGTGATGGATGATAATGGAGCTGCTGTATATGAAGAGATGCCAGTGGACTCCTGGAAGATGCCGTATACCAACAGACACAAGCGGAGTCCTAAACGCTGCAAGTTTTGCTGTAATTGCTGTCCTGGAATGCGGGGATGTGGTGTCTGCTGCAGGTTCTGA | >TrhampII-4 XM_029838810.1  MKTFSVAVVAAVLLALICLQESSALPLSEVEDVEVPVMDDNGAA VYEEMPVDSWKMPYTNRHKRSPKRCKFCCNCCPGMRGCGVCCRF |
|  |  | TrhampII-5: XM_003965899.3 |  | 8153422-8154212 | >TrhampII-5 XM_003965899.3  ATGAAGACCTTCAGTGTTGCTGTTGTTGCGGCCGTCCTGCTGGCCCTCATTTGTCTCCAGGAGAGCTCTGCTCTTCCTCTCAGTGAATTGGAAGATGTGGAGGTGCCAGTGATGGATGATAATGGAGCTGCTGTATATGAAGAGATGCCAGTGGACTCCTGGAAGATGCCGTATACCAACAGACACAAGCGGAGTCCTAAACGCTGCAAGTTTTGCTGTAATTGCTGTCCTGGAATGCGGGGATGTGGTGTCTGCTGCAGGTTCTGA | >TrhampII-5: XM_003965899.3  MKTFSVAVVAAVLLALICLQESSALPLSELEDVEVPVMDDNGAAVYEEMPVDSWKMPYTNRHKRSPKRCKFCCNCCPGMRGCGVCCR |
|  |  | TrhampII-6: XM_029838809.1 |  | 8156569-8157360 | >TrhampII-6 XM_029838809.1  ATGAAGACCTTCAGTGTTGCTGTTGTTGCGGCCGTCCTGCTGGCCCTCATTTGTCTCCAGGAGAGCTCTGCTCTTCCTCTCAGTGAATTGGAAGATGTGGAGGTGCCAGTGATGGATGATAATGGAGCTGCTGTATATGAAGAGATGCCAGTGGACTCCTGGAAGATGCCGTATACCAACAGACACAAGCGGAGTCCTAAACGCTGCAAGTTTTGCTGTAATTGCTGTCCTGGAATGCGGGGATGTGGTGTCTGCTGCAGGTTCTGA | >TrhampII-6 XM_029838809.1  MKTFSVAVVAAVLLALICLQESSALPLSELEDVEVPVMDDNGAAVYEEMPVDSWKMPYTNRHKRSPKRCKFCCNCCPGMRGCGVCCR |
|  |  | TrhampII-7: XM-003979040.3 |  | 8158818-8159608 | >TrhampII-7 XM_029838809.1  ATGAAGACCTTCAGTGTTGCTGTTGTTGCGGCCGTCCTGCTGGCCCTCATTTGTCTCCAGGAGAGCTCTGCTCTTCCTCTCAGTGAATTGGAAGATGTGGAGGTGCCAGTGATGGATGATAATGGAGCTGCTGTATATGAAGAGATGCCAGTGGACTCCTGGAAGATGCCGTATACCAACAGACACAAGCGGAGTCCTAAACGCTGCAAGTTTTGCTGTAATTGCTGTCCTGGAATGCGGGGATGTGGTGTCTGCTGCAGGTTCTGA | >TrhampII-7 XM_029838809.1  MKTFSVAVVAAVLLALICLQESSALPLSELEDVEVPVMDDNGAAVYEEMPVDSWKMPYTNRHKRSPKRCKFCCNCCPGMRGCGVCCRF |
|  |  | TrhampII-8: XM_029839113.1 |  | 8164800-8165411 | >TrhampII-8 XM_029839113.1  ATGAAGACCTTCAGTGTTGCTGTTGTTGCGGCCGTCCTGCTGGCCCTCATTTGTCTCCAGGAGAGCTCTGCTCTTCCTCTCAGTGAATTGGAAGATGTGGAGGTGCCAGTGATGGATGATAATGGAGCTGCTGTATATGAAGAGATGCCAGTGGACTCCTGGAAGATGCCGTATACCAACAGACACAAGCGGAGTCCTAAACGCTGCAAGTTTTGCTGTAATTGCTGTCCTGGAATGCGGGGATGTGGTGTCTGCTGCAGGTTCTGA | >TrhampII-8 XM_029839113.1  MKTFSVAVVAAVLLALICLQESSALPLSELEDVEVPVMDDNGAAVYEEMPVDSWKMPYTNRHKRSPKRCKFCCNCCPGMRGCGVCCRF |
|  |  | TrhampII-9: XM-029839114.1 |  | 8173304-8177369 | >TrhampII-9 XM_029839114.1  ATGAAGACCTTCAGTGTTGCTGTTGTTGCGGCCGTCCTGCTGGCCCTCATTTGTCTCCAGGAGAGCTCTGCTCTTCCTCTCAGTGAAGTGGAAGATGTGGAGGTGCCAGTGATGGATGATAATGGAGCTGCTGTATATGAAGAGATGCCAGTGGACTCCTGGACGATGCCGTATACCAACAGACACAAGCGGAGTCCTAAACGCTGCAGGTTTTGCTGTAATTGCTGTCCTAGAATGGTGGGATGTGGTACCTGCTGCAAGTTC | >TrhampII-9 XM_029839114.1  MKTFSVAVVAAVLLALICLQESSALPLSEVEDVEVPVMDDNGAAVYEEMPVDSWTMPYTNRHKRSPKRCRFCCNCCPRMVGCGTCCKF |
| *Lates calcarifer* | GCA_001640805.1 | LcalhampII-1: XM_018683350.1 | NW_017363830.1 | 3446145-3447316 | >LcalhampII-1 XM_018683350.1  ATGAAGACATTCAGTGTTGCAGTTGCAGTGGCCGTCGTGCTCGCCATCATTTGCATTCAGGAGAGTTCTGCCTTCACATTCACTGGGGTACAAGAGCTGGAGGAGCTAATGAGCAATGACAATCCAGTTGCTGAACATGAAGAGACAGCAGTGGATTCATGGATGATGCCATATAACTTCAGACAGAAGCGTCAGAGCGGCCCTGCTGCAAAAAAGTGCCGTTTGTGCTGTAACTGTTGCCCAGGGATGAGAGGCTGCGGTGTTTGCTGCAAATTCTGA | >LcalhampII-1 XM_018683350.1  MKTFSVAVAVAVVLAIICIQESSAFTFTGVQELEELMSNDNPVAEHEETAVDSWMMPYNFRQKRQSGPAAKKCRLCCNCCPGMRGCGVCCKF |
|  |  | LcalhampII-2: XM_018683362.1 |  | 3449788-3450693 | >LcalhampII-2 XM_018683362.1  ATGAAGACATTCAGTGTTGCAGTTGCAGTGGCCGTCGTGCTCGCCATCATTTGCATTCAGGAGAGTTCTGCCTTCACATTCACTGGGGTACAAGAGCTGGAGGAGCTAATGAGCAATGACAATCCAGTTGCTGAACATGAAGAGACAGCAGTGGATTCATGGATGATGCCATATAACTTCAGACAGAAGCGTCAGAGCGGCCCTGCTGCAAAAAAGTGCCGTTTGTGCTGTAACTGTTGCCCAGGGATGAGAGGCTGCGGTGTTTGCTGCAAATTCTGA | >LcalhampII-2: XM_018683362.1  MKTFSVAVAVAVVLAIICIQESSAFTFTGVQELEELMSNDNPVAEHEETAVDSWMMPYNFRQKRQSGPAAKKCRLCCNCCPGMRGCGVCCKF |
|  |  | LcalhampII-3: XM_018683387.1 |  | 3453431-3454285 | >LcalhampII-3 XM_018683387.1  ATGAAGACATTCAGTGTTGCAGTTGCAGTGGCCGTCGTGCTCGCCATCATTTGCATTCAGGAGAGTTCTGCCTTCACATTCACTGGGGTACAAGAGCTGGAGGAGCTAATGAGCAATGACAATCCAGTTGCTGAACATGAAGAGACAGCAGTGGATTCATGGATGATGCCATATAACTTCAGACAGAAGCGTCAGAGCGGCCCTGCTGCAAAAAAGTGCCGTTTGTGCTGTAACTGTTGCCCAGGGATGAGAGGCTGCGGTGTTTGCTGCAAATTCTGA | >LcalhampII-3: XM_018683387.1  MKTFSVAVAVAVVLAIICIQESSAFTFTGVQELEELMSNDNPVAEHEETAVDSWMMPYNFRQKRQSGPAAKKCRLCCNCCPGMRGCGVCCKF |
|  |  | LcalhampII-4: XM_018683371.1 |  | 3457077-3457931 | >LcalhampII-4 XM_018683371.1  ATGAAGACATTCAGTGTTGCAGTTGCAGTGGCCGTCGTGCTCGCCATCATTTGCATTCAGGAGAGTTCTGCCTTCACATTCACTGGGGTACAAGAGCTGGAGGAGCTAATGAGCAATGACAATCCAGTTGCTGAACATGAAGAGACAGCAGTGGATTCATGGATGATGCCATATAACTTCAGACAGAAGCGTCAGAGCGGCCCTGCTGCAAAAAAGTGCCGTTTGTGCTGTAACTGTTGCCCAGGGATGAGAGGCTGCGGTGTTTGCTGCAAATTCTG | >LcalhampII-4 XM_018683371.1  MKTFSVAVAVAVVLAIICIQESSAFTFTGVQELEELMSNDNPVAEHEETAVDSWMMPYNFRQKRQSGPAAKKCRLCCNCCPGMRGCGVCCKF |
|  |  | LcalhampII-5: XM_018683415.1 |  | 3460715-3461634 | >LcalhampII-5 XM_018683415.1  ATGAAGACATTCAGTGTTGCAGTTGCAGTGGCCGTCGTGCTCGCCATCATTTGCATTCAGGAGAGTTCTGCCTTCACATTCACTGGGGTACAAGAGCTGGAGGAGCTAATGAGCAATGACAATCCAGTTGCTGAACATGAAGAGACAGCAGTGGATTCATGGATGATGCCATATAACTTCAGACAGAAGCATCAGAGCGGCCCTGCTGCAAAAAAGTGCCGTTTGTGCTGTAACTGTTGCTCAGGGATGAGAGGCTGCGGTGTTTGCTGCAAATTCTGA | >LcalhampII-5 XM_018683415.1  MKTFSVAVAVAVVLAIICIQESSAFTFTGVQELEELMSNDNPVAEHEETAVDSWMMPYNFRQKHQSGPAAKKCRLCCNCCSGMRGCGVCCKF |
|  |  | LcalhampII-6: XM_018683401.1 |  | 3464352-3465300 | >LcalhampII-6 XM_018683401.1  ATGAAGACATTCAGTGTTGCAGTTGCAGTGGCCGTCATGCTTGCCATCATTTCTCTTGAAGAGAGTTCTGCCTTCACATTCACTGGGGTACAAGAGCTGGAGGAGCTAATGAGCAATGACAATCCAGTTGCTGAACATGAAGAGACAGCAGTGGATTCATGGATGATGCCATATAACTTCAGACAGAAGCGTCAGAGCGGCCCTGCTGCAAAAAAGTGCCGTTTGTGCTGTAACTGTTGCCCAGGGATGAGAGGCTGCGGTGTTTGCTGCAAATTCTGA | >LcalhampII-6 XM_018683401.1  MKTFSVAVAVAVMLAIISLEESSAFTFTGVQELEELMSNDNPVAEHEETAVDSWMMPYNFRQKRQSGPAAKKCRLCCNCCPGMRGCGVCCKF |
| *Oreochromis niloticus* | GCA_001858045.3 | OnhampII-1: XM_025911618.1 | LG11  NC_031976.2 | 13823939-13830029 | >OnhampII-1 XM_025911618.1  ATGAAGACGTTCAGTGCTGCAGTTGCAGTGGCCGTCGTGCTCACATTCATCTGTGTTCAGCAGAGCTCTGCTGTCCCAGTCACTGAACTGGAGGAGCCAATGAGCATGGACTATCCAGCAGCAGCACCTGAGCAGATATTAGTGGACTCATGGAAGAAGTTGTATAACACCATACACAGGCCTGGCATCAAGTGTCGCTTTTGCTGTGGCTGCTGCACCCCCGGTATCTGTGGAATTTGCTGCAGTAAAC | >OnhampII-1 XM_025911618.1  MKTFSAAVAVAVVLTFICVQQSSAVPVTELEEPMSMDYPAAAPEQILVDSWKKLYNTIHRPGIKCRFCCGCCTPGICGICCSKLL |
|  |  | OnhampII-2: XM_025911268.1 |  | 13818291-13821669 | >OnhampII-2 XM_025911268.1  ATGAAGACGTTCAGTGTTGCAGTTGCAGTGGCCGTCGTGCTCACATTCATCTGTGTTCAGCAGAGCTCTGCTGTCCCAGTCACTGAAGAGCTGGAGGAGCCAATGAGCATGGACTATCCAGCAGCAGCACATGAGGAGGCATCAGTGGACTCATGGAAGATGCTGTATAACAGCAGACACAAGCGTGGCATCAAGTGTCGCTTTTGCTGTGGCTGCTGCACCCCCGGTATCTGTGGAGTTTGCTGCAGGTTCTGA | >OnhampII-2 XM_025911268.1  MKTFSVAVAVAVVLTFICVQQSSAVPVTEELEEPMSMDYPAAAHEEASVDSWKMLYNSRHKRGIKCRFCCGCCTPGICGVCCRF |
|  |  | OnhampII-3: XM_019365032.2 |  | 13778687-13780491 | >OnhampII-3 XM_019365032.2  ATGAAGACGTTCAGTGTTGCAGTTGCAGTGGCCGTCGTGCTCACATTCATCTGTGTTCAGCAGAGCTCTGCTGTCCCAGTCACTGAAGAGCTGGAGGAGCCAATGAGCATGGACTATCCAGCAGCAGCACATGAGGAGGCATCAGTGGACTCATGGAAGATGCTGTATAACAGCAGACACAAGCGTGGCATCAAGTGTCGCTTTTGCTGTGGCTGCTGCACCCCCGGTATCTGTGGAGTTTGCTGCAGGTTCTGA | >OnhampII-3 XM_019365032.2  MKTFSVAVAVAVVLTFICVQQSSAVPVTEELEEPMSMDYPAAAHEEASVDSWKMLYNSRHKRGIKCRFCCGCCTPGICGVCCRF |
|  |  | OnhampII-4: XM_019365117.2 |  | 13762863-13763721 | >OnhampII-4 XM_019365117.2  ATGAAGACGTTCAGTGTTGCAGTTGCAGTGGCCGTCGTGCTCACATTCATCTGTGTTCAGCAGAGCTCTGCTGTCCCAGTCACTGAAGAGCAGGAGCTGGAGGAGCCAATGAGCATGGACTATCCAGCAGCAGCACATGAGGAGGCATCAGTGGACTCATGGAAGATGCTGTATAACAGCAGACACAAGCGTGGCATCAAGTGTCGCTTTTGCTGTGGCTGCTGCACCCCCGGTATCTGTGGAGTTTGCTGCAGGTTCTGA | >OnhampII-4 XM_019365117.2  MKTFSVAVAVAVVLTFICVQQSSAVPVTEEQELEEPMSMDYPAAAHEEASVDSWKMLYNSRHKRGIKCRFCCGCCTPGICGVCCRF |
|  |  | OnhampII-5: XM_019365119.2 |  | 13756685-13757482 | >OnhampII-5 XM_019365119.2  ATGAAGACGTTCAGTGTTGCAGTTGCAGTGGCCGTCGTGCTCACATTCATCTGTGTTCAGCAGAGCTCTGCTGTCCCAGTCACTGAACAGGAGCAGGAGCTGGAGGAGCCAATGAGCATGGACTATCCAGCAGCAGCACATGAGGAGGCATCAGTGGACTCATGGAAGATGCTGTATAACAGCAGACACAAGCGTGGCATCAAGTGTCGCTTTTGCTGTGGCTGCTGCACCCCTGGTATCTGTGGAGTTTGCTGCAGGTTCTGA | >>OnhampII-5 XM_019365119.2  MKTFSVAVAVAVVLTFICVQQSSAVPVTEQEQELEEPMSMDYPAAAHEEASVDSWKMLYNSRHKRGIKCRFCCGCCTPGICGVCCRF |
|  |  | OnhampII-6: XM_025911266.1 |  | 13750451-13751256 | >OnhampII-6 XM_025911266.1  ATGAAGACGTTCAGTGTTGCAGTTGCAGTGGCCGTCGTGCTCACATTCATCTGTGTTCAGCAGAGCTCTGCTGTCCCAGTCACTGAAGAGCTGGAGGAGCCAATGAGCATGGACTATCCAGCAGCAGCACATGAGGAGGCATCAGTGGACTCATGGAAGATGCTGTATAACAGCAGACACAAGCGTGGCATCAAGTGTCGCTTTTGCTGTGGCTGCTGCACCCCCGGTATCTGTGGAGTTTGCTGCAGGTTCTGA | >OnhampII-6 XM_025911266.1  MKTFSVAVAVAVVLTFICVQQSSAVPVTEELEEPMSMDYPAAAHEEASVDSWKMLYNSRHKRGIKCRFCCGCCTPGICGVCCRF |
|  |  | OnhampII-7: XM_005450674.3 |  | 13734628..13735355 | >OnhampII-7 XM_005450674.3  ATGAAGACACTGACAGCTGCAGTTACAGTGGCCATTGTGGTCACATTCATGTGTATTCTGAGGAGCTCTGCTGTCCCTGTTGTTGAAGAGCAAATGCTGGTGGAGCTAATGAACACTGACAATCCAGCTGCAGAACCTGAAGAGATATCAGTGAACTCGTGGAAGATGCTGCACAATGGAAGACAAAAGCGTGGTATCTTTTGCGGCAGGTGCTGTAATGGCAAAGTCTGCTGGAGGTGCTGCTATGCCTAA | >OnhampII-7 XM_005450674.3  MKTLTAAVTVAIVVTFMCILRSSAVPVVEEQMLVELMNTDNPAAEPEEISVNSWKMLHNGRQKRGIFCGRCCNGKVCWRCCYA |
| *Gasterosteus aculeatus* | GCA_016920845.1 | GahampII-1: XM_040164187.1 | NC_053231.1 | 15567201-15567960 | >GahampII-1 XM_040164187.1  ATGAAGACATTCAGTGTTGCAGTTGCAGTCGCCATCGTGCTCACCTTCATCTGTATCCAGCAGAGCTCTGCTGTCCCAACAGCTGAGGTGCAGGAAGTGGAGGAGCCAATGAGTGTTGAGTTCCCACTTGCTGAGCAGGAGGAGACATCAGTGGACTCCTGGAAGATGCCGTATAACATCAGAGAGAAGCGCGGCATCAAGTGCAAGTTTTGCTGTGGCTGCTGCACCCCCGGGGTCTGTGGATTGTGCTGCAGATTCTGA | >GahampII-1 XM_040164187.1  MKTFSVAVAVAIVLTFICIQQSSAVPTAEVQEVEEPMSVEFPLAEQEETSVDSWKMPYNIREKRGIKCK  FCCGCCTPGVCGLCCRF |
|  |  | GahampII-2: XM_040165533.1 |  | 15564044-15564819 | >GahampII-2 XM_040165533.1  ATGAAGACATTCAGTGTTGCAGTTGCAGTCGCCATCGTGCTCACCTTCATCTGTATCCAGCAGAGCTCTGCTGTCCCAACAGCTGAGGTGCAGGAAGTGGAGGAGCCAATGAGTGTTGAGTTCCCACTTGCTGAGCAGGAAGAGACATCAGTGGACTCCTGGAAGATGCCGTATAACATCAGAGAGAAGCGCGGCATCAAGTGCAAGTTTTGCTGTGGCTGCTGCACCCCCGGGGTCTGTGGATTGTGCTGCAGATTCTGA | >GahampII-2 XM_040165533.1  MKTFSVAVAVAIVLTFICIQQSSAVPTAEVQEVEEPMSVEFPLAEQEETSVDSWKMPYNIREKRGIKCKFCCGCCTPGVCGLCCRF |
|  |  | GahampII-3: XM_040164189.1 |  | 15560157-15560915 | >GahampII-3 XM_040164189.1  ATGAAGACATTCAGTGTTGCAGTTGCAGTCGCCATCGTGCTCACCTTCATCTGTATCCAGCAGAGCTCTGCTGTCCCAACAGCTGAGGTGCAGGAAGTGGAGGAGCCAATGAGTGTTGAGTTCCCACTTGCTGAGCAGGAAGAGACATCAGTGGACTCGTGGATGATGCCGTATAACATCAGAGAGAAGCGCGGCATCAAGTGCAAGTTTTGCTGTGGCTGCTGCACCCCCGGGGTCTGTGGATTGTGCTGCAGATTCTGA | >GahampII-3 XM_040164189.1  MKTFSVAVAVAIVLTFICIQQSSAVPTAEVQEVEEPMSVEFPLAEQEETSVDSWMMPYNIREKRGIKCKFCCGCCTPGVCGLCCRF |
| *Larimichthys crocea* | GCA_000972845.2 | LcrohampII-1: ENSLCRT00005018573.1 | NC_040023.1 | 25190050-25190597 | >LcrohampII-1 ENSLCRT00005018573.1  ATGAAGACATTCAGTGTTGCAGTTGCAGTGGCCGTCATGCTCGCCTTCATTTGTCTTCAGGAGAGCTCTGCTGTCCCAGTCAATGAAGAGCAAGAGCTGGAGCAGCAAATTTATTTTGATGATCCAGAGATGCCAGTGGAATCATGGAAGATGCCGTATCACCTGCGACAGAAGCGTCACAGCAGCGCCGCTAAATGCAAGTTTTGTTGCAGATGCTGTCCTAGAATGACTGGATGTGGTGTCTGCTGCAGGTTC | >LcrohampII-1 ENSLCRT00005018573.1  MKTFSVAVAVAVMLAFICLQESSAVPVNEEQELEQQIYFDDPEMPVESWKMPYHLRQKRHSSAAKCKFCCRCCPRMTGCGVCCRF |
|  |  | LcrohampII-2: ENSLCRT00005018598.1 |  | 25195094-25195637 | >LcrohampII-2 ENSLCRT00005018598.1  ATGAAGACATTCAGTGTTGCAGTTGCAGTGGCCGTCATGCTCGCCTTCATTTGTCTTCAGGAGAGCTCTGCTGTCCCAGTCAATGAAGAGCAAGAGCTGGAGCAGCAAATTTATTTTGCTGATCCAGAGATGCCAGTGGAATCATGGAAGATGCCGTATCACCTGCGACAGAAGCGTCACAGCAGCGCCGCTAAATGCAAGTTTTGTTGCGGTTGCTGTCCTAGAATGTCTGGATGTGGTGTCTGCTGCAGGTTC | >LcrohampII-2 ENSLCRT00005018598.1  MKTFSVAVAVAVMLAFICLQESSAVPVNEEQELEQQIYFADPEMPVESWKMPYHLRQKRHSSAAKCKFCCGCCPRMSGCGVCCRF |
|  |  | LcrohampII-3: XM_027286856.1 |  | 25198085_25198885 | >LcrohampII-3 XM_027286856.1  ATGAAGACATTCAGTGTTGCAGTTGCAGTGGCCGTCATGCTCGCCTTCATTTGTCTTCAGGAGAGCTCTGCTGTCCCAGTCAATGAAGAGCAAGAGCTGGAGCAGCAAATTTATTTTGATGATCCAGAGATGCCAGTGGAATCATGGAAGATGCCGTATCACCTGCGACAGAAGCGTCACAGCAGCGCCGCTAAATGCAAGTTTTGTTGCAGATGCTGTCCTAGAATGACTGGATGTGGTGTCTGCTGCAGGTTC | >LcrohampII-3 XM_027286856.1  MKTFSVAVAVAVMLAFICLQESSAVPVNEEQELEQQIYFDDPEMPVESWKMPYHLRQKRHSSAAKCKFCCRCCPRMTGCGVCCRF |
|  |  | LcrohampII-4: XM_027286857.1 |  | 25200094_25200950 | >LcrohampII-4 XM_027286857.1  ATGAAGACATTCAGTGTTGCAGTTGCAGTGGCCGTCATGCTCGCCTTCATTTGTCTTCAGGAGAGCTCTGCTGTCCCAGTCAATGAAGAGCAAGAGCTGGAGCAGCAAATTTATTTCGCTGATCCAGAGATGCCAGTGGAATCATGGAAGATGCCGTATCACCTGCGACAGAAGCGTCACAGCAGCGCCGCTAAATGCCTGTTTTGTTGCAGATGCTGTCCTGATATGATTGGATGTGGTATCTGCTGCAGGTTC | >LcrohampII-4 XM_027286857.1  MKTFSVAVAVAVMLAFICLQESSAVPVNEEQELEQQIYFADPEMPVESWKMPYHLRQKRHSSAAKCLFCCRCCPDMIGCGICCRF |
|  |  | LcrohampII-5: XM_027286855.1 |  | 25202027_25202828 | >LcrohampII-5 XM_027286855.1  ATGAAGACATTCAGTGTTGCAGTTGCAGTGGCCGTCATGCTCGCCTTCATTTGTCTTCAGGAGAGCTCTGCTGTCCCAGTCAATGAAGAGCAAGAGCTGGAGCAGCAAATTTATTTTGATGATCCAGAGATGCCAGTGGAATCATGGAAGATGCCGTATCACCTGCGACAGAAGCGTCACAGCAGCGCCGCTAAATGCAAGTTTTGTTGCAGATGCTGTCCTAGAATGACTGGATGTGGTGTCTGCTGCAGGTTC | >LcrohampII-5 XM_027286855.1  MKTFSVAVAVAVMLAFICLQESSAVPVNEEQELEQQIYFDDPEMPVESWKMPYHLRQKRHSSAAKCKFCCRCCPRMTGCGVCCRF |
|  |  | LcrohampII-6: XM_027286858.1 |  | 25204045_25204901 | >LcrohampII-6 XM_027286858.1  ATGAAGACATTCAGTGCTGCAGTTGCAATGGCCGTCATGCTCGCCTTCATTTGTCTTCAGGAGAGCTCTGCTGTCCCAGTCACTGAAGAGCAAGAGCTGGAGCAGCACATTTATTTTGATGATCCAGAGATGCCAGTGGAATCATGGAAGATGCCGTATCACCTGCGACAGAAGCGTCACAGCAGCGCCGCTAAATGCAAGTTTTGTTGCAGATGCTGTCCTGATATGATTGGATGTGGTGTCTGCTGCAGGTTC | >LcrohampII-6 XM_027286858.1  MKTFSAAVAMAVMLAFICLQESSAVPVTEEQELEQHIYFDDPEMPVESWKMPYHLRQKRHSSAAKCKFCCRCCPDMIGCGVCCRF |
| *Lepisosteus oculatus* | GCA_000242695.1 | LohampI: XM_006641649.2 | LG24 ;  NC_023202 | 3519815-3522716 | >LohampI XM_006641649.2  ATGAAGGCTCTGTCTGTTGCGGTGTTGGTCGTGCTGCTGTCAGTCTGTATTCAAAGCAGCGACGCTGTCCCATTCGCAGAGGCAGAAGTACAGGAGACAGAGGCTGAACATAGTAGTCCAGCAGAGGTACAGATGAATTTTGCTGATGAGGAAGTGCAATCACTGACTGAAGGCAAGTTGCGGACAAAGAGGCAAAGTCACCTTTCTCTCTGCAGATACTGCTGCAATTGCTGTCATAACAAAGGCTGTGGATTCTGCTGTAGATTTTAG | >LohampI XM_006641649.2  MKALSVAVLVVLLSVCIQSSDAVPFAEAEVQETEAEHSSPAEVQMNFADEEVQSLTEGKLRTKRQSHLSLCRYCCNCCHNKGCGFCCRF- |

### Table S2. dN/dS value of antimicrobial peptides in the lined seahorse

(Excel table)

### Table S3. Primers used for amplifying coding sequences and qPCR analysis of seahorse HehampIIs

| Gene name | Primer sequences (Forward and Reverse) (5′→3′) |
| --- | --- |
| Primers used for amplifying coding sequence | |
| *HehampII-1* | F: ATGAAGCCCTTCAGTTTGTCT |
|  | R: TCACCAGTCACAGCACATAC |
| *HeHampII-2* | F: ATGAAGCCCTTCAGTTTGTC |
|  | R: TCAATATTCGCAGCAAAAGG |
| *HehampII-3* | F: ATGAAGCCCTTCAGTTTTTCT |
|  | R: TCACCATTCGCAGCACAAGCC |
| *HehampII-4* | F: ATGAAGCCCTTCAGTTTGTC |
|  | R: TTAGAATCTCCATTCGCAGC |
| Primers used for qPCR analysis | |
| *HehampII-1* | F: TGGAAGCAAGAGATGACGCA |
|  | R: TGTCGATTCTCGACCACAGC |
| *HehampII-2* | F: TAGGGAGCCTGACCAGCATA |
|  | R: TCAATATTCGCAGCAAAAGG |
| *HehampII-3* | F: TGGCGGAAACAAGAGAAGACGC |
|  | R: CAAGCCACAGAAATTTGTTTTA |
| *HehampII-4* | F: GGTGGAAACAAGAGAAGACGC |
|  | R: TTAGAATCTCCATTCGCAGC |
| *β-actin* | F: TTCACCACCACAGCCGAGA |
|  | R: TGGTCTCGTGGATTCCGCAG |

### Table S4. Information on the chemically synthesised seahorse HeHampII mature peptides

| AMPs | Synthesised amino acid sequence of HampII mature peptide | Molecular weight (Dalton) | Purity (HPLC) |
| --- | --- | --- | --- |
| HeHampII-2 | HNRGCRFCCNCCGRTHFCAFCCEY | 2833.6 | 95.6% |
| HeHampII-4 | HSGPCKFCCNCCGRMHFCGFCCEWRF | 3062.4 | 96.1% |
| PC-hepc | QGSPARCRFCCRCCPRMRGCGICCRF | 2970.1 | 96.5% |

### Table S5. Microbial agglutination of seahorse HeHampII mature peptides

|  |  | HeHampII-2 | | | | | | HeHampII-4 | | | | | |
| --- | --- | --- | --- | --- | --- | --- | --- | --- | --- | --- | --- | --- | --- |
| Strains | Control | 3 μM  (8.5  μg/mL) | 6 μM  (17  μg/mL) | 12 μM  (34  μg/mL) | 24 μM  (68  μg/mL) | 48 μM  (136  μg/mL | 96 μM  (272  μg/mL | 3 μM  (9.2  μg/mL) | 6 μM  (18.37  μg/mL) | 12 μM  (36.75  μg/mL) | 24 μM  (73.50  μg/mL) | 48 μM  (147  μg/mL) | 96 μM  (294  μg/mL) |
| **Gram-positive bacteria** |  |  |  |  |  |  |  |  |  |  |  |  |  |
| *Bacillus subtilis* | — | + | ++ | +++ | +++ | +++ | +++ | + | ++ | +++ | +++ | +++ | +++ |
| *Bacillus thuringiensis* | — | + | ++ | +++ | +++ | +++ | +++ | + | ++ | +++ | +++ | +++ | +++ |
| *Micrococcus luteus* | — | + | ++ | +++ | +++ | +++ | +++ | + | ++ | +++ | +++ | +++ | +++ |
| *Staphylococcus aureus* | — | + | ++ | +++ | +++ | +++ | +++ | + | ++ | +++ | +++ | +++ | +++ |
| *Enterococcus faecalis* | — | + | ++ | +++ | +++ | +++ | +++ | + | ++ | +++ | +++ | +++ | +++ |
| Methicillin-resistant *Staphylococcus aureus (MRSA)* | — | + | ++ | +++ | +++ | +++ | +++ | + | ++ | +++ | +++ | +++ | +++ |
| Methicillin-Resistant *Staphylococcus epidermidis* (MRSE) | — | + | ++ | +++ | +++ | +++ | +++ | + | ++ | +++ | +++ | +++ | +++ |
| **Gram-negative bacterium** |  |  |  |  |  |  |  |  |  |  |  |  |  |
| *Vibrio parahaemolyticus* | — | + | ++ | +++ | +++ | +++ | +++ | + | ++ | +++ | +++ | +++ | +++ |
| *Escherichia coli* | — | + | ++ | +++ | +++ | +++ | +++ | + | ++ | +++ | +++ | +++ | +++ |
| *Acinetobacter baumannii* | — | + | ++ | +++ | +++ | +++ | +++ | + | ++ | +++ | +++ | +++ | +++ |
| *Klebsiella pneumoniae* | — | + | ++ | +++ | +++ | +++ | +++ | + | ++ | +++ | +++ | +++ | +++ |
| *Salmonella typhimurium* | — | + | ++ | +++ | +++ | +++ | +++ | + | ++ | +++ | +++ | +++ | +++ |
| **Fungi** |  |  |  |  |  |  |  |  |  |  |  |  |  |
| *Candida albicans* | — | + | ++ | +++ | +++ | +++ | +++ | + | ++ | +++ | +++ | +++ | +++ |

+++: approximate 95% – 100% bacterial cell agglutination

++: approximate 70% – 95% bacterial cell agglutination

+: approximate 30% – 70% bacterial cell agglutination

—: No visible agglutination

## 3 Information for qPCR experiments


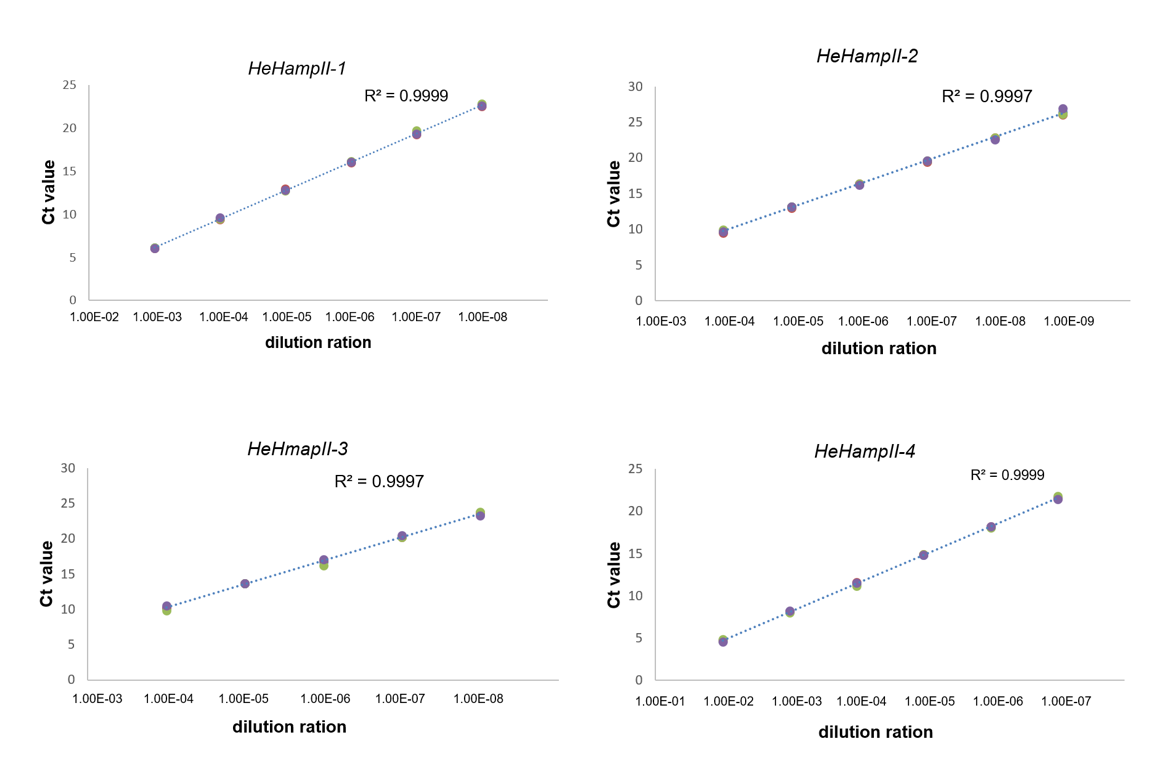


### Figure S5. Curves show values for the dilution ration of plasmid plotted against Ct values from qRT-PCR amplification.


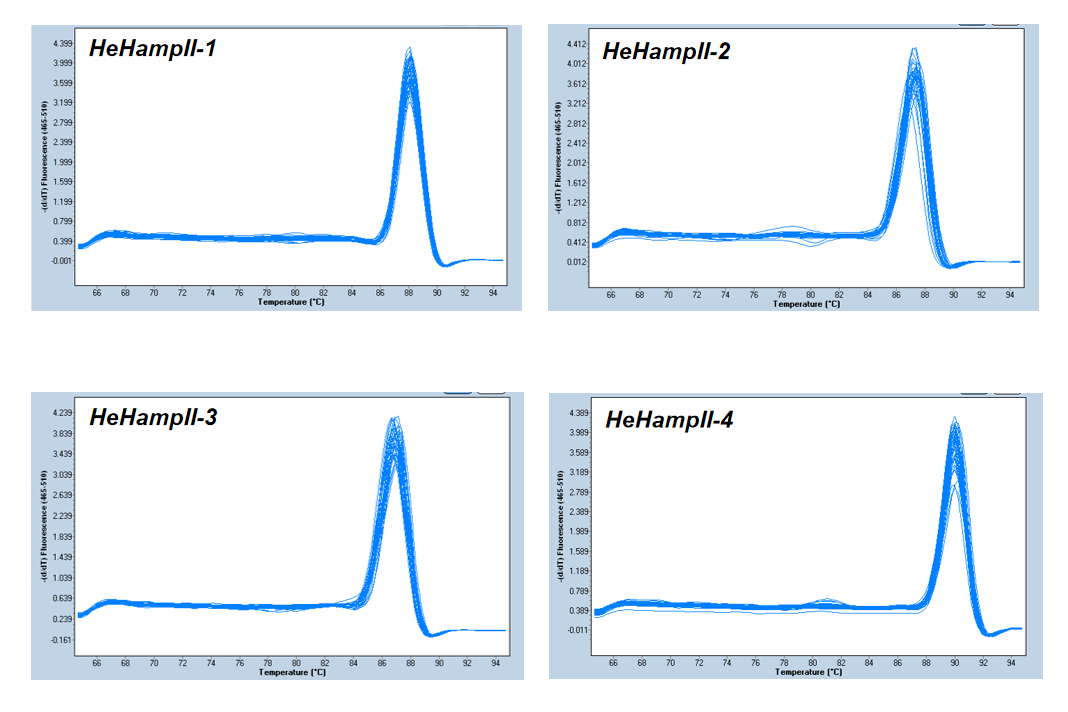


### Figure S6. The melting curve excluded nonspecific amplification products.

### Table S6. qPCR amplification system

| Composition of reaction | Volume (μL) |
| --- | --- |
| H_2_O | 3 |
| SYBR Green Real-time PCR | 5 |
| Sense primer (10 μM) | 0.5 |
| Antisense primer (10 μM) | 0.5 |
| cDNA | 1 |
| Total volume | 10 |
